# VdOMO contributes to development, stress adaptation, siderophore-associated iron acquisition, and early host colonization in *Verticillium dahliae*

**DOI:** 10.3389/fpls.2026.1905175

**Published:** 2026-08-19

**Authors:** Yusha Du, Lixinyu Sun, Kangwei Xie, Jiatong Zhang, Wei Jian, Niqi Xie, Xingyong Yang

**Affiliations:** 1 College of Pharmacy, Chengdu University, Chengdu, China; 2 School of Life Science, Chongqing Normal University, Chongqing, China; 3 Minxian No. 2 Middle School of Gansu Province, Min County, Gansu, China; 4 Department of Laboratory Medicine, The Affiliated Dazu’s Hospital of Chongqing Medical University, Chongqing, China

**Keywords:** iron acquisition, L-ornithine N5-monooxygenase, SidA, siderophore, stress adaptation, *Verticillium dahliae*

## Abstract

*Verticillium dahliae* is a soilborne fungal pathogen that causes Verticillium wilt in cotton and many other crops. While siderophore-associated iron acquisition is recognized as crucial, its precise connections to fungal development, stress adaptation, and host colonization remain incompletely elucidated. This study identifies the VdOMO protein as a putative SidA-family L-ornithine N5-monooxygenase through phylogenetic and pairwise sequence analyses. Analyses of independent deletion mutants and a complemented strain showed that *VdOMO* contributes to conidial morphology and production, microsclerotium formation, and melanization, growth on selected carbon sources, cell wall integrity, and adaptation to alkaline, salt, and oxidative stresses. Although final visible disease symptoms were comparable among inoculated groups, *ΔVdOMO* mutants exhibited reduced vascular browning, diminished fungal biomass accumulation at early infection stages, and impaired cellophane penetration. A representative *ΔVdOMO* mutant also displayed decreased biomass-normalized extracellular chrome azurol S (CAS)-reactive iron-chelating activity and intracellular iron accumulation, both of which were restored to wild-type levels upon complementation. Furthermore, *VdOMO* deletion was linked to iron-condition-dependent alterations in the expression of genes involved in iron regulation, siderophore biosynthesis, and siderophore transport. Collectively, these findings underscore a significant role for *VdOMO* in siderophore-associated iron acquisition, fungal development, stress adaptation, and the early stages of host colonization in *V. dahliae*.

## 1 Introduction

The genus *Verticillium* comprises soilborne ascomycetes that cause vascular wilt diseases in a wide array of crops (Fradin and Thomma, 2006; Jeseničnik et al., 2023). *Verticillium dahliae*, a prominent species within this genus, is a major causal agent of Verticillium wilt, infecting over 400 dicotyledonous species and leading to substantial yield losses, particularly in cotton (Song et al., 2020; Dadd-Daigle et al., 2021). The long-term persistence of *V. dahliae* in soil is largely attributed to its melanized microsclerotia, where 1,8-dihydroxynaphthalene (DHN) melanin provides crucial protection for survival under adverse environmental conditions (Bell et al., 1976; Xiong et al., 2019).

*Verticillium dahliae* infects plants through their roots, penetrating epidermal and cortical tissues before colonizing the xylem vessels. This extensive vascular colonization disrupts water and nutrient transport, leading to wilting, vascular discoloration, and overall plant decline (Shaban et al., 2018; Song et al., 2020). Successful infection by *V. dahliae* is a complex process involving coordinated steps such as microsclerotium germination, root adhesion, hyphal penetration, xylem colonization, nutrient acquisition, stress adaptation, and modulation of host defense responses (Song et al., 2020; Zhu et al., 2023). Numerous *V. dahliae* genes and signaling pathways are involved in these processes, including regulators of microsclerotium formation and melanin biosynthesis (Hu et al., 2014; Harting et al., 2020; Wen et al., 2023), MAPK- and stress-related pathways (Turrà et al., 2014; Yu et al., 2019; Wang et al., 2023), infection structure development (Zhao et al., 2016; Bui et al., 2019), and iron homeostasis (Wang et al., 2018). These findings suggest that *V. dahliae* pathogenicity is not determined by a single virulence process but is closely associated with fungal development, environmental adaptation, nutrient acquisition, and host colonization. Given that host colonization requires adaptation to plant-derived nutrients, studying fungal growth on relevant carbon substrates can provide insights into nutrient utilization during host interaction. Sucrose is a major soluble carbohydrate in plant tissues, while pectin is a principal component of plant cell walls. Xylose and galactose are representative monosaccharides derived from plant cell-wall polysaccharides (Koch, 2004; Cosgrove, 2005; Voragen et al., 2009). Therefore, these substrates were chosen to evaluate whether *VdOMO* affects growth on plant-relevant carbon sources.

Iron acquisition and homeostasis are crucial for fungal nutrient metabolism, stress adaptation, and pathogenicity. Although iron is essential for fungal growth, development, and host colonization, its bioavailability is frequently limited in natural and host environments. Consequently, many filamentous fungi rely on siderophore-mediated pathways to acquire iron and maintain intracellular iron balance (Pecoraro et al., 2021). Fungal L-ornithine N5-monooxygenases (OMO) catalyze an early, committed step in the biosynthesis of hydroxamate-type siderophores (Hissen et al., 2005; Pecoraro et al., 2021). Homologous proteins with this conserved enzymatic function are known as SidA, Sid1, Sib2, or OMO1 in various fungal species (Mei et al., 1993; Mercier and Labbé, 2010; Plante and Labbé, 2019). For instance, in *Aspergillus fumigatus*, SidA is indispensable for siderophore biosynthesis and virulence (Schrettl et al., 2004; Hissen et al., 2005). SidA-family homologs are also found in phytopathogenic fungi, model filamentous fungi, basidiomycetes, and yeast, though their broader contributions to fungal development and environmental adaptation are not yet fully understood. In *V. dahliae*, iron homeostasis has been linked to fungal development, stress adaptation, and virulence (Wang et al., 2018). Therefore, we selected *VDAG_05313*, hereafter referred to as *VdOMO*, to investigate whether this predicted SidA-family L-ornithine N5-monooxygenase connects siderophore-associated iron acquisition with fungal development and stress adaptation.

*VdOMO* is located in the same genomic region as *VDAG_05314* and *VdASP* (*VDAG_05317*). *VdASP* encodes a predicted L-asparaginase containing an Asparaginase_2 domain; however, its transcriptional relationship with *VdOMO* has not been established in *V. dahliae*. Therefore, these genes were included in a time-course expression analysis to evaluate possible expression-level associations without assuming direct co-regulation or functional interaction.

In this study, we generated independent *VdOMO* deletion mutants and complemented strains to investigate the contributions of *VdOMO* to fungal development, carbon-source utilization, stress adaptation, cell wall integrity, iron acquisition, and cotton colonization. We further quantified extracellular CAS-reactive iron-chelating activity and intracellular iron content under different iron-availability conditions, examined the expression of iron-related genes, and evaluated the expression-level association among *VdOMO*, *VDAG_05314*, and *VdASP*.

## 2 Materials and methods

### 2.1 Fungal strains, plant material, and culture conditions

*Verticillium dahliae* strain Vd991 served as the wild-type (WT) parental strain for generating the Vd991-derived *VdOMO* deletion mutants and complemented strains used in this study. Cotton (*Gossypium hirsutum* L.) cultivar ‘Yumian 1’ was used for the infection assays. A previously generated and molecularly validated *ΔVdASP-1* mutant derived from Vd991 was used exclusively for the time-course transcriptional analysis. Fungal strains were preserved in 50% glycerol at −80 °C and cultured on potato-dextrose agar (PDA), potato-dextrose broth (PDB), or Czapek-Dox medium, unless otherwise specified. Czapek-Dox medium contained 30 g/L sucrose, 2 g/L NaNO_3_, 1 g/L K_2_HPO_4_, 0.5 g/L MgSO_4_·7H_2_O, 0.5 g/L KCl, and 0.01 g/L FeSO_4_·7H_2_O; 20 g/L agar was added for solid medium. Minimal medium (MM) contained 6 g/L NaNO_3_, 1.52 g/L KH_2_PO_4_, 0.52 g/L KCl, 0.52 g/L MgSO_4_·7H_2_O, and 20 mM L-glutamic acid; 15 g/L agar was added for solid MM. MM was supplemented with Fe or bathophenanthroline disulfonate (BPS), as indicated. Strains used for the quantitative liquid CAS assay were cultured in agar-free low-iron MM. Routine phenotypic assays, including those for vegetative growth, microsclerotium formation, stress response, cell wall integrity, and penetration, were performed at 26 °C unless otherwise specified. Iron-related assays were conducted at 25 °C.

### 2.2 Conserved-domain, phylogenetic, and pairwise sequence analyses

Conserved regions in the VdOMO protein were analyzed using the NCBI Conserved Domain Database (CDD) (Lu et al., 2020). Representative full-length fungal L-ornithine N5-monooxygenase homologs annotated as SidA, Sid1, Sib2, OMO1, or SidA-like proteins were retrieved from the NCBI Protein and UniProtKB databases (The UniProt, C, 2021), and their accession numbers are listed in **Supplementary Table 1**. Amino acid sequences were aligned using MAFFT v7 (Katoh and Standley, 2013). A maximum-likelihood tree was inferred using IQ-TREE v2.2.0 (Minh et al., 2020) under the LG+F+G4 model selected by ModelFinder according to the Bayesian information criterion (Kalyaanamoorthy et al., 2017), with 1,000 ultrafast bootstrap replicates. The unrooted maximum-likelihood topology was midpoint-rooted solely for visualization and displayed as a cladogram.

Pairwise local alignments between VdOMO and representative homologs were performed using PairwiseAligner module in Biopython v1.86 (Cock et al., 2009), with the BLOSUM62 substitution matrix and gap-opening and gap-extension penalties of 10 and 0.5, respectively. Identity was calculated from identical residue pairs within non-gap aligned positions, whereas similarity was determined from residue pairs with positive BLOSUM62 scores. Coverage was calculated as the proportion of VdOMO residues included in each alignment (**Supplementary Table 1**).

### 2.3 Generation and molecular validation of *VdOMO* and *VdASP* mutants in *V. dahliae*

Gene deletion and complementation were performed using *Agrobacterium tumefaciens*-mediated transformation (ATMT). Approximately 1.5-kb 5′ and 3′ flanking regions of *VdOMO* were amplified from Vd991 genomic DNA using primer pairs VdOMO-5OUT/VdOMO-5R and VdOMO-3F/VdOMO-3OUT, respectively. The hygromycin-resistance cassette (*hph*) was amplified from pSilent-1 using hyg-F/hyg-R (**Supplementary Table 2**). The three fragments (5′ flank, *hph* cassette, and 3′ flank) were fused by overlap PCR. The resulting fragment was seamlessly cloned into XbaI/BamHI-linearized pkoxn. This construct was then introduced into *A. tumefaciens* AGL-1 and subsequently transferred into Vd991 via ATMT. Transformants were selected on PDA containing 30 μg/mL hygromycin and further purified on PDA supplemented with 30 μg/mL hygromycin and 500 μg/mL cefotaxime. Deletion transformants were verified by locus-spanning PCR (VdOMO-5OUT/VdOMO-3OUT), 5′-junction PCR (VdOMO-5OUT/hyg-R), 3′-junction PCR (hyg-F/VdOMO-3OUT), and *hph*-specific PCR (hyg-F/hyg-R). *ΔVdOMO-7* was selected as the representative deletion mutant for subsequent transcriptional and iron-related analyses and is hereafter referred to as *ΔVdOMO*.

For complementation, a 4.6-kb genomic fragment containing *VdOMO* was amplified using VdOMOCom-F/VdOMOCom-R and assembled into EcoRI/BamHI-linearized pCOM. This construct was introduced into the *ΔVdOMO* background through ATMT, and transformants were selected on PDA containing 30 μg/mL geneticin. Restoration of *VdOMO* transcription was confirmed by RT-PCR, using 18S rRNA as a cDNA-quality control. Among the validated transformants, *C-ΔVdOMO-3* was used as the complemented strain in phenotypic and iron-related assays and is hereafter referred to as *C-ΔVdOMO*.

The *ΔVdASP* mutants were generated previously in our laboratory from Vd991 by ATMT. Approximately 1.5-kb 5′ and 3′ flanking regions of *VdASP* (*VDAG_05317*) were amplified using VdASP-5OUT/VdASP-5R and VdASP-3F/VdASP-3OUT, respectively, and fused with the hygromycin-resistance cassette (*hph*). The resulting replacement fragment was assembled into the pkoxn vector and introduced into Vd991. Hygromycin-resistant transformants were screened, and replacement of the native *VdASP* locus was verified by locus-spanning PCR using VdASP-5OUT/VdASP-3OUT, *hph*-specific PCR using hyg-F/hyg-R, 5′-junction PCR using VdASP-5OUT/hyg-R, and 3′-junction PCR using hyg-F/VdASP-3OUT (**Supplementary Table 2**; **Supplementary Figures 1a, b**). Loss of *VdASP* transcription was further confirmed by RT-PCR using 18S rRNA as the cDNA-quality control (**Supplementary Figure 1c**). Two independent deletion mutants, *ΔVdASP-1* and *ΔVdASP*-*2*, were obtained. *ΔVdASP-1* was selected for time-course transcriptional analysis and is hereafter referred to as *ΔVdASP*.

### 2.4 Phenotypic observations and conidial production

Conidial suspensions of Vd991, *ΔVdOMO-7*, *ΔVdOMO-9*, and *C-ΔVdOMO* were adjusted to 1 × 10^7^ spores/mL. A 3 μL aliquot of each conidial suspension was inoculated onto antibiotic-free PDA plates, which were then sealed and incubated in an inverted position at 26 °C for 7 ad.

Vd991 and the representative *ΔVdOMO-7* mutant were cultured in PDB containing the corresponding antibiotics at 26 °C and 180 rpm for 5–7 d. Conidia were collected and examined by scanning electron microscopy.

A 1 mL aliquot of each conidial suspension was added to 100 mL of antibiotic-free PDB and incubated at 26 °C and 180 rpm for 7 ad. Following incubation, mycelia were removed by filtration, and conidial concentrations in the filtrates were quantified. Three independent biological replicates were performed. Transcriptional analysis of three conidiation-related genes, *VdNLP1* (*VDAG_04701.1*), *VdPLP* (*VDAG_00942*), and *Vdpf* (*VDAG_08521.1*), was conducted by RT-qPCR using *Histone3* as the internal reference gene (**Supplementary Table 3**). Unless otherwise specified, all phenotypic assays described in Sections 2.5-2.9 were performed on antibiotic-free media.

### 2.5 Assessment of microsclerotium formation and melanization

Conidial suspensions of Vd991, *ΔVdOMO-7*, *ΔVdOMO-9*, and *C-ΔVdOMO* were adjusted to 1 × 10^7^ spores/mL and inoculated onto CM. Cultures were incubated at 26 °C and monitored for microsclerotium development and melanization. Mycelial samples containing developing microsclerotia were collected with a sterile toothpick and examined microscopically. Each experiment was conducted in triplicate. To further assess transcriptional changes, 11 microsclerotium- and melanization-associated genes (*VDAG_06474*, *VDAG_02474*, *VDAG_08521*, *VDAG_00192*, *VDAG_06686*, *VDAG_03674*, *VDAG_00184*, *VDAG_03393*, *VDAG_00190*, *VDAG_00183*, and *VDAG_03665*) were analyzed by RT-qPCR using *Histone3* as the internal reference gene (**Supplementary Table 3**) (Hu et al., 2014; Harting et al., 2020; Wen et al., 2023).

### 2.6 Carbon source utilization assays

Czapek-Dox medium was prepared with sucrose, pectin, xylose, or galactose as the sole carbon source. Conidial suspensions of Vd991, *ΔVdOMO-7*, *ΔVdOMO-9*, and *C-ΔVdOMO* were inoculated onto the respective media in 90-mm Petri dishes. Colony diameters were measured after incubation at 26 °C for 7 ad.

### 2.7 Environmental stress assays

To evaluate the responses of the *VdOMO* deletion mutants to pH, salt, osmotic, and oxidative stresses, PDA was prepared at pH 5, 7, 9, 11, and 13. CM medium served as the basal medium, to which varying concentrations of stress agents were added: 0.8 M NaCl, 1 M NaCl, 1.2 M NaCl, 1 M sorbitol, and 1.2 M sorbitol. Spore suspensions of Vd991, *ΔVdOMO-7*, *ΔVdOMO-9*, and *C-ΔVdOMO* strains were prepared, with a spore concentration adjusted to 1 × 10^7^ spores/mL. A 3 μL aliquot of each prepared spore suspension was added dropwise to the center of a plate, which was then sealed with a film and incubated inverted at 26 °C.

For the oxidative stress assay, CM solid medium was prepared by autoclaving at 121 °C for 20 minutes. After cooling to approximately 50 °C, 1 mL of spore suspension from the Vd991, *ΔVdOMO-7*, *ΔVdOMO-9*, and *C-ΔVdOMO* strains was added, thoroughly mixed, and then uniformly poured into Petri dishes in a sterile environment. After solidification, sterile 0.5-cm-diameter filter-paper discs were placed at the center of the medium using sterile forceps. Subsequently, 5 µL of 5% and 15% H_2_O_2_ were added to the center of the filter paper, and the dish was sealed and incubated inverted at 26 °C for 3–4 d. After the formation of inhibition zones, the sizes of the zones were measured, photographed, and recorded.

### 2.8 Cell wall integrity analysis

CM medium, autoclaved at 121 °C for 20 min, served as the base. After cooling, Congo red was aseptically added to a final concentration of 300 μg/mL, and the medium was poured into 60-mm Petri dishes. Spore suspensions of Vd991, *ΔVdOMO-7*, *ΔVdOMO-9*, and *C-ΔVdOMO* strains were prepared and adjusted to a concentration of 1 × 10^7^ spores/mL. A 3 μL aliquot of each prepared spore suspension was pipetted onto the center of a plate, which was then sealed with sealing film and incubated at 26 °C.

### 2.9 Assays for cotton infection, fungal colonization, and mycelial penetration

Cotton infection and fungal colonization were assessed using the susceptible cotton cultivar ‘Yumian 1’ at the two-true-leaf stage. The wounded root inoculation method was employed, where each pot received 30 mL of a conidial suspension of the indicated strain at 2 × 10^8^ spores/mL. Each strain treatment comprised 24 pots in each of three independent experiments, totaling 72 pots per strain. The mock control, consisting of the same number of pots, received sterile water. Whole-plant symptoms were photographed at 21 days post-inoculation (dpi). Vascular browning and fungal recovery were assessed at 20 dpi. For vascular browning assessment, the root–stem junction of each inoculated cotton plant was excised and longitudinally sectioned using a sterile razor blade. The browning of the cotton vascular bundles was examined and photographically documented using a stereoscopic optical microscope. Concurrently, stems of the inoculated cotton plants were cut into approximately 3 cm segments, rinsed with sterile water, and disinfected with a 3% sodium hypochlorite solution for 5 min. Following this, the segments were rinsed again with sterile water and surface disinfected with 75% alcohol for 1 min. The stems were then cut into smaller segments (approximately 5 mm) using sterilized scissors, placed on PDA, and monitored for fungal regrowth.

Cotton roots were collected at 9, 12, and 15 dpi. Fungal genomic DNA was extracted using the TIANGEN DNAsecure novel plant genomic DNA extraction kit to quantify *V. dahliae* biomass via qPCR. The SYBRPRIME qPCR Kit (Fast HS) was used, with the cotton *GhUBQ7* gene as the internal reference and ITS as the target region (**Supplementary Table 3**). Mycelial penetration differences among Vd991, *ΔVdOMO-7*, *ΔVdOMO-9*, and *C-ΔVdOMO* strains were evaluated by adding conidial suspensions (2 × 10^6^ spores/mL) dropwise to the centers of sterile cellophane membranes placed on MM medium. After incubation at 26 °C for 3 ad, membranes were removed, and incubation continued for an additional 4 ad. Photographs were taken once the WT colony covered the plate. Three independent biological replicates were performed.

To assess associated transcriptional changes, mycelia of Vd991 and the representative *ΔVdOMO-7* mutant were collected after 5 and 10 ad of growth. Transcript levels of the penetration- and colonization-associated genes *VdNoxB*, *VdPls1*, *VdCrz1*, *VdSep5*, *VdCSIN1*, *Vta3*, and *Som1* were quantified by RT-qPCR using *Histone3* as the internal reference gene (**Supplementary Table 3**) (Zhao et al., 2016; Bui et al., 2019).

### 2.10 Expression analysis of defense-related genes in cotton

RNA was extracted from cotton stems at 3, 6, 9, 12, and 15 dpi after inoculation with Vd991 or the representative *ΔVdOMO-7* mutant. Total RNA was reverse-transcribed into cDNA to assess the expression of cotton disease-response genes, including *NPR1*, *PR1*, *OPR3*, *PR5*, *ERF1*, and *PDF1.2*. Additionally, SA-related genes (*ICS1*, *EDS1*, and *PAD4*) and JA-related genes (*AOC1*, *AOC2*, and *TCP)* were analyzed (**Supplementary Table 3**). The *Histone3* gene served as the internal reference, and the WT sample collected at 3 dpi was used as the calibrator. Relative transcript levels were calculated using the 2^−ΔΔCt method.

### 2.11 Effects of iron availability on growth and conidial production

Strains were cultivated on PDA or MM at 25 °C. For iron-related treatments, MM was used alone or supplemented with 0.03 mM Fe_2_(SO_4_)_3_, equivalent to 0.06 mM Fe, or 0.4 mM bathophenanthroline disulfonate (BPS). For dry-biomass and conidial-yield assays, strains were cultured in liquid MM with or without 0.03 mM Fe_2_(SO_4_)_3_. Dry biomass and conidial yield were determined after 14 ad and 7 ad of cultivation, respectively.

### 2.12 Quantification of extracellular CAS-reactive iron-chelating activity

Extracellular CAS-reactive iron-chelating activity was quantified using a liquid chrome azurol S (CAS) shuttle assay, based on the method by Schwyn and Neilands (1987) and adapted to a 96-well microplate format as described by Arora and Verma (2017). Vd991, *ΔVdOMO*, and *C-ΔVdOMO* were inoculated into 50 mL of agar-free low-iron liquid MM lacking Fe_2_(SO_4_)_3_, FeSO_4_, and bathophenanthroline disulfonate (BPS) at 1 × 10^6^ spores/mL and incubated at 25 °C and 200 rpm for 72 ah.

Culture supernatants were collected by centrifugation at 10,000 × g for 10 min and filtered through 0.22-μm membrane filters. Equal volumes of filtered supernatant and freshly prepared CAS shuttle reagent (100 μL each) were mixed in 96-well microplates, incubated in the dark at room temperature for 60 min, and measured at 630 nm. Uninoculated low-iron liquid MM was used as the reference. Background-corrected absorbance values were obtained using paired wells containing supernatant or low-iron liquid MM mixed with deionized water. CAS-reactive iron-chelating activity was calculated as [(Ar − As)/Ar] × 100, where Ar and As represent the background-corrected absorbance values of the reference and sample reactions, respectively. The corresponding mycelia were washed, dried at 60 °C to a constant weight, and used to normalize the CAS-reactive activity, which was expressed as % CAS units mg^−1^ dry biomass.

### 2.13 Quantification of intracellular iron content

To assess the effect of *VdOMO* on intracellular iron accumulation, the WT, representative *ΔVdOMO-7* mutant, and representative *C-ΔVdOMO-3* strain were cultured in MM, MM+Fe, or MM+BPS. Mycelia were harvested, thoroughly washed, dried to a constant weight, and used for cellular iron quantification. Intracellular iron content was determined using a Cell Iron Content Assay Kit (BC5315; Solarbio, Beijing, China) according to the manufacturer’s instructions. This assay involves the reduction of ferric iron to ferrous iron, which then reacts with o-phenanthroline under weakly acidic conditions to form an orange-red complex. Absorbance was measured at 510 nm using a microplate reader. Iron content was calculated based on a standard curve and normalized to mycelial dry biomass, with data expressed as ng mg^−1^ dry biomass.

### 2.14 Transcriptional analysis of *VdOMO*, *VDAG_05314*, and *VdASP*

Total RNA was extracted from Vd991, the representative *ΔVdOMO-7* mutant, and *ΔVdASP-1* after 7, 10, and 13 ad of cultivation using Orizol RNA Extraction Reagent (Oriscience Biotechnology Co., Shanghai, China). cDNA was synthesized using the PrimeScript RT Reagent Kit (Takara Bio Inc., Dalian, China), and RT-qPCR was performed using the Tianlong Gentier 96R system with SYBR Green. Transcript levels of *VdOMO*, *VDAG_05314*, and *VdASP* were quantified using the primers listed in **Supplementary Table 3**. The *β-tubulin* gene served as the internal reference, and the WT sample collected at 7 ad was used as the calibrator. Relative transcript levels were calculated using the 2^−ΔΔCt method (Livak and Schmittgen, 2001).

### 2.15 Expression analysis of iron-related genes

The WT, representative *ΔVdOMO-7* mutant, and representative *C-ΔVdOMO-3* strain were initially grown in liquid MM at 25 °C and 200 rpm for 3 ad. Subsequently, they were transferred to fresh MM, MM+Fe, or MM+BPS for 45 min. Mycelia were immediately collected and frozen in liquid nitrogen. RNA extraction, cDNA synthesis, and RT-qPCR were performed as described in Section 2.14. Transcript levels of *HapX*, *srbA*, *sreA*, *sidD*, *mirB*, *mirA1*, *mirA2*, and *VDAG_08042* were quantified using *β-tubulin* as the internal reference gene and WT cultured in MM as the calibrator (**Supplementary Table 3**). Relative transcript levels were calculated using the 2^−ΔΔCt method (Livak and Schmittgen, 2001).

### 2.16 Statistical analysis

All quantitative experiments incorporated a minimum of three independent biological replicates. When technical replicates were employed, their mean was considered a single value for each biological replicate, ensuring technical replicates were not treated as independent observations. Data are presented as mean ± standard deviation (SD). Normality and homogeneity of variance were evaluated using the Shapiro–Wilk and Levene’s tests, respectively. All datasets subjected to parametric analyses satisfied these assumptions (*P* > 0.05). For comparisons between groups, an unpaired two-tailed Student’s *t*-test was utilized. Single-factor experiments involving three or more groups were analyzed using one-way ANOVA followed by Tukey’s multiple-comparison test. Two-factor experiments were analyzed using two-way ANOVA, with either Tukey’s or Šídák’s multiple-comparison test applied as specified in the corresponding figure legends. Statistical analyses of RT-qPCR data were performed using ΔCt values, while relative expression levels are presented as 2^−ΔΔCt values. A *P* value < 0.05 was considered statistically significant. Statistical analyses were conducted using SPSS v27.0 and GraphPad Prism v10.0.2.

## 3 Results

### 3.1 Sequence features and phylogenetic placement of *VdOMO*

**Figure 1:**
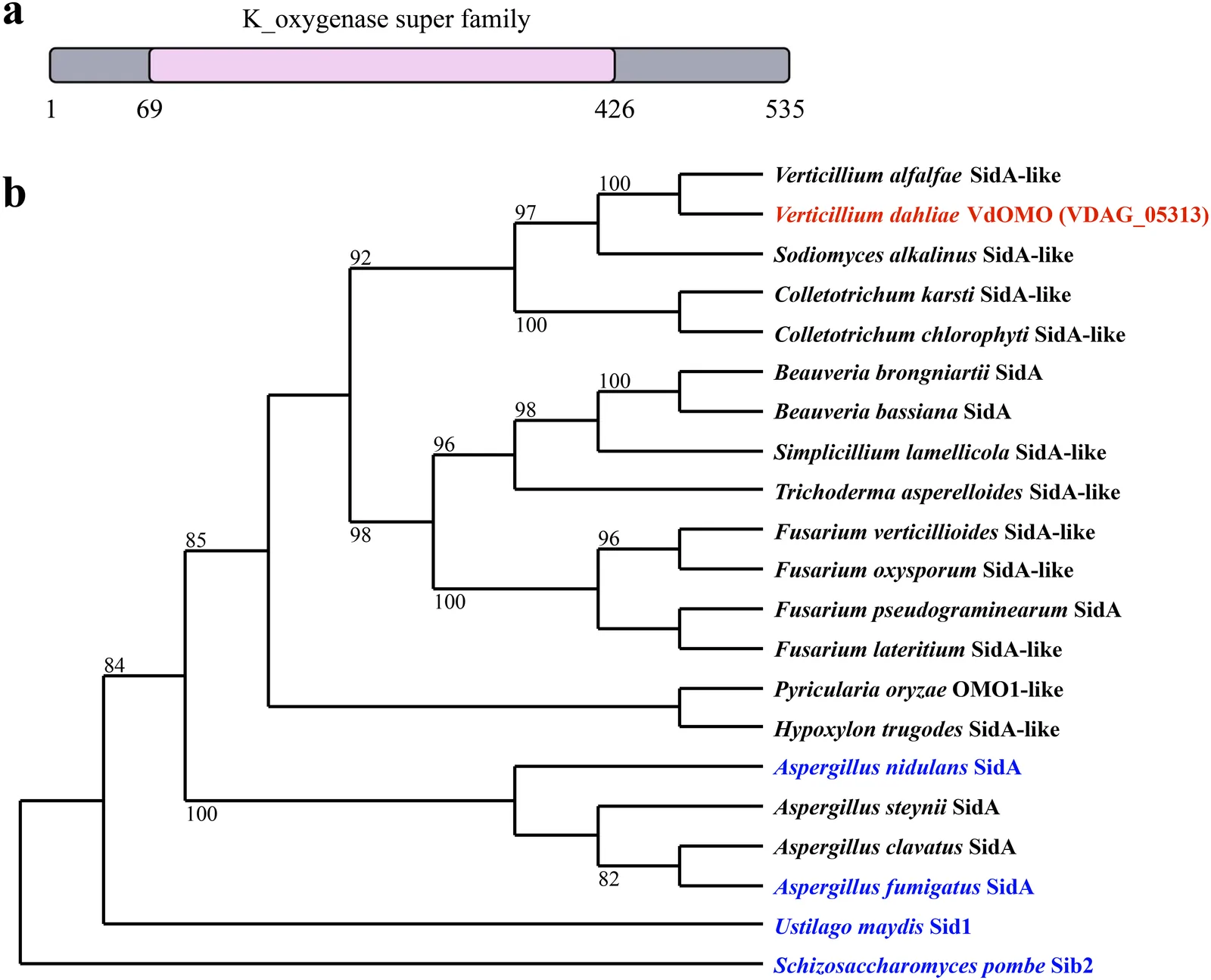
Conserved-domain architecture and phylogenetic placement of VdOMO. **(a)** CDD-predicted region belonging to the K_oxygenase superfamily at residues 69–426 of VdOMO. **(b)** Midpoint-rooted maximum-likelihood cladogram of VdOMO and representative fungal SidA/Sid1/Sib2/OMO1 homologs. The tree was inferred from MAFFT-aligned full-length amino acid sequences using IQ-TREE v2.2.0 under the LG+F+G4 model. The maximum-likelihood topology was initially inferred as an unrooted tree and midpoint-rooted solely for visualization. Ultrafast bootstrap support was evaluated using 1,000 replicates, with only values ≥80% shown. VdOMO is highlighted in red, while *Aspergillus nidulans* SidA, *A. fumigatus* SidA, *Ustilago maydis* Sid1, and *Schizosaccharomyces pombe* Sib2 are highlighted in blue. Branch lengths are not proportional to sequence divergence as the tree is displayed as a cladogram. Accession numbers and pairwise identity, similarity, and coverage values are provided in **Supplementary Table 1**.

The *VDAG_05313* gene, designated *VdOMO*, contains a 1,608-bp open reading frame that encodes a 535-amino-acid protein. Conserved Domain Database (CDD) analysis identified a conserved region spanning residues 69-426, which belongs to the K_oxygenase superfamily (**Figure 1a**). Phylogenetic analysis positioned VdOMO within the fungal SidA/Sid1/Sib2/OMO1 homologous protein group. Notably, VdOMO formed a strongly supported sister relationship with the *Verticillium alfalfae* SidA-like protein (ultrafast bootstrap support = 100%; **Figure 1b**). The dataset also included homologs from the model fungi *Aspergillus nidulans* and *Ustilago maydis*, the pathogenic fungus *A. fumigatus*, and the fission yeast *Schizosaccharomyces pombe*.

Pairwise local alignments showed amino acid identities ranging from 38.72% to 98.58%, similarity from 60.84% to 99.19%, and VdOMO sequence coverage from 86.54% to 99.81% across the selected homologs (**Supplementary Table 1**). The highest conservation was observed with the *V. alfalfae* SidA-like protein, which shared 98.58% identity, 99.19% similarity, and 98.50% VdOMO sequence coverage. These findings support the classification of VdOMO as a putative SidA-family L-ornithine N5-monooxygenase.

### 3.2 Generation and molecular validation of *VdOMO* and *VdASP* mutants

**Figure 2:**
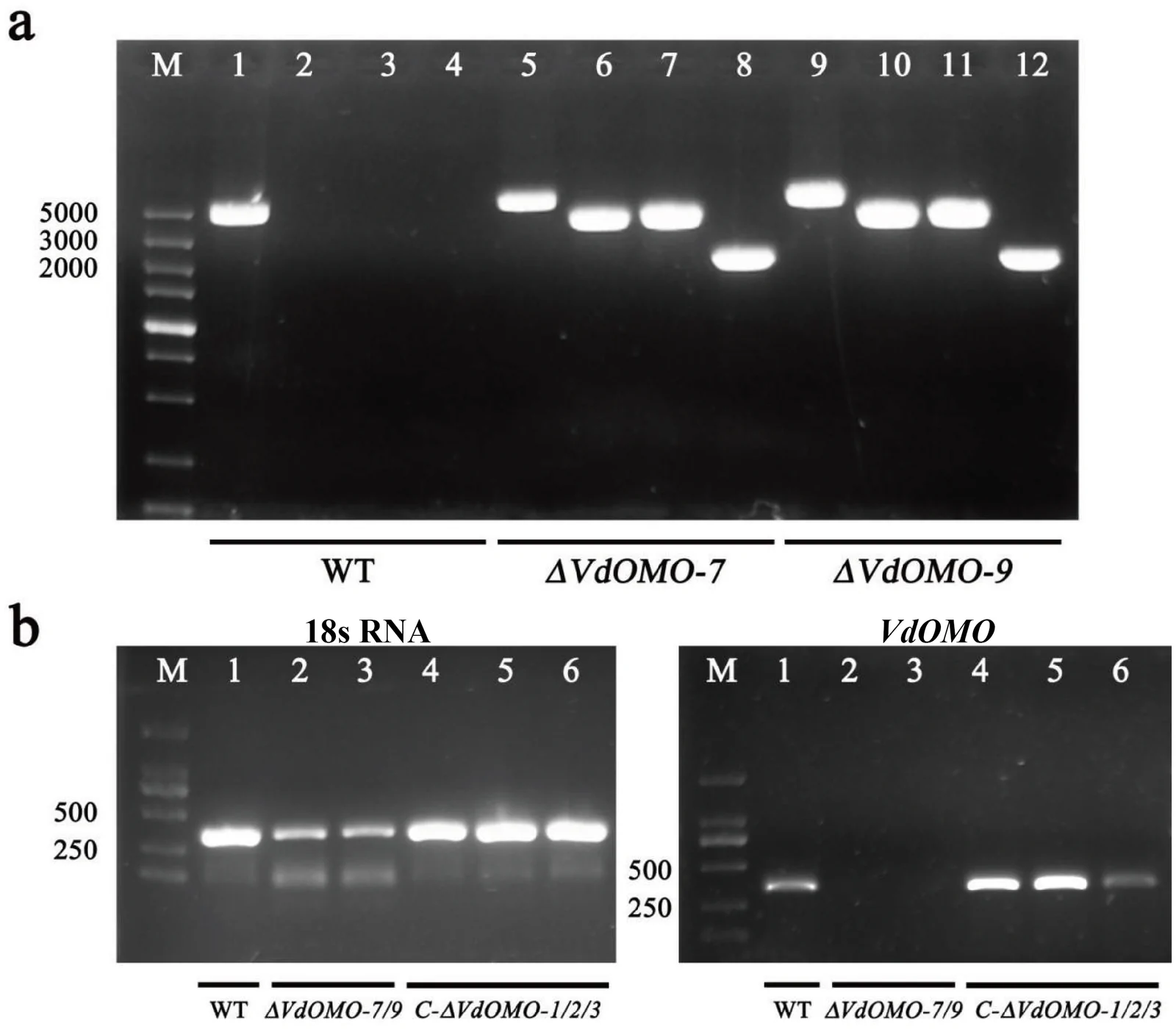
Molecular validation of the *VdOMO* deletion and complemented strains. **(a)** Diagnostic PCR of WT, *ΔVdOMO-7*, and *ΔVdOMO-9*. M, DNA marker; lanes 1-4, WT; lanes 5-8, *ΔVdOMO-7*; lanes 9-12, *ΔVdOMO-9*. Lanes 1, 5, and 9, locus-spanning PCR; lanes 2, 6, and 10, 5′ recombination junction; lanes 3, 7, and 11, 3′ recombination junction; lanes 4, 8, and 12, *hph* cassette. **(b)** RT-PCR detection of *VdOMO* transcripts. Lanes 1-6, WT, *ΔVdOMO-7*, *ΔVdOMO-9*, *C-ΔVdOMO-1*, *C-ΔVdOMO-2*, and *C-ΔVdOMO-3*, respectively. The 18S rRNA amplicon served as the cDNA-quality control.

Two independent deletion mutants, *ΔVdOMO-7* and *ΔVdOMO-9*, were generated from the parental strain Vd991. PCR analysis confirmed the successful replacement of the native *VdOMO* locus with the *hph* cassette. Locus-spanning PCR yielded larger amplicons in both deletion mutants compared to the WT strain, consistent with the insertion of the *hph* cassette. Furthermore, expected 5’- and 3’-junction products, along with the *hph*-specific product, were detected in both deletion mutants but not in the WT (**Figure 2a**).

Three complemented strains (*C-ΔVdOMO-1*, *C-ΔVdOMO-2*, and *C-ΔVdOMO-3*) were subsequently obtained. RT-PCR confirmed the presence of *VdOMO* transcripts in the WT and complemented strains, while no transcripts were detected in *ΔVdOMO-7* or *ΔVdOMO-9*. The 18S rRNA control was detected across all strains, validating cDNA quality (**Figure 2b**). These findings collectively confirm the successful deletion and complementation of *VdOMO*. Based on comparable molecular validation and subsequent phenotypic characterization, *ΔVdOMO-7* was selected as the representative deletion mutant for further transcriptional and iron-related analyses. Similarly, *C-ΔVdOMO-3* was designated as the representative complemented strain for iron-related analyses, unless otherwise specified.

Additionally, two previously generated *VdASP* deletion mutants, *ΔVdASP-1* and *ΔVdASP-2*, underwent molecular validation. Diagnostic PCR confirmed the presence of expected locus-spanning, *hph*-specific, and 5’- and 3’-recombination-junction products in both mutants. RT-PCR demonstrated *VdASP* transcripts in the WT but not in either deletion mutant (**Supplementary Figure 1**). Consequently, *ΔVdASP-1* was chosen for the time-course transcriptional analysis.

### 3.3 *VdOMO* contributes to conidial morphology and production

**Figure 3:**
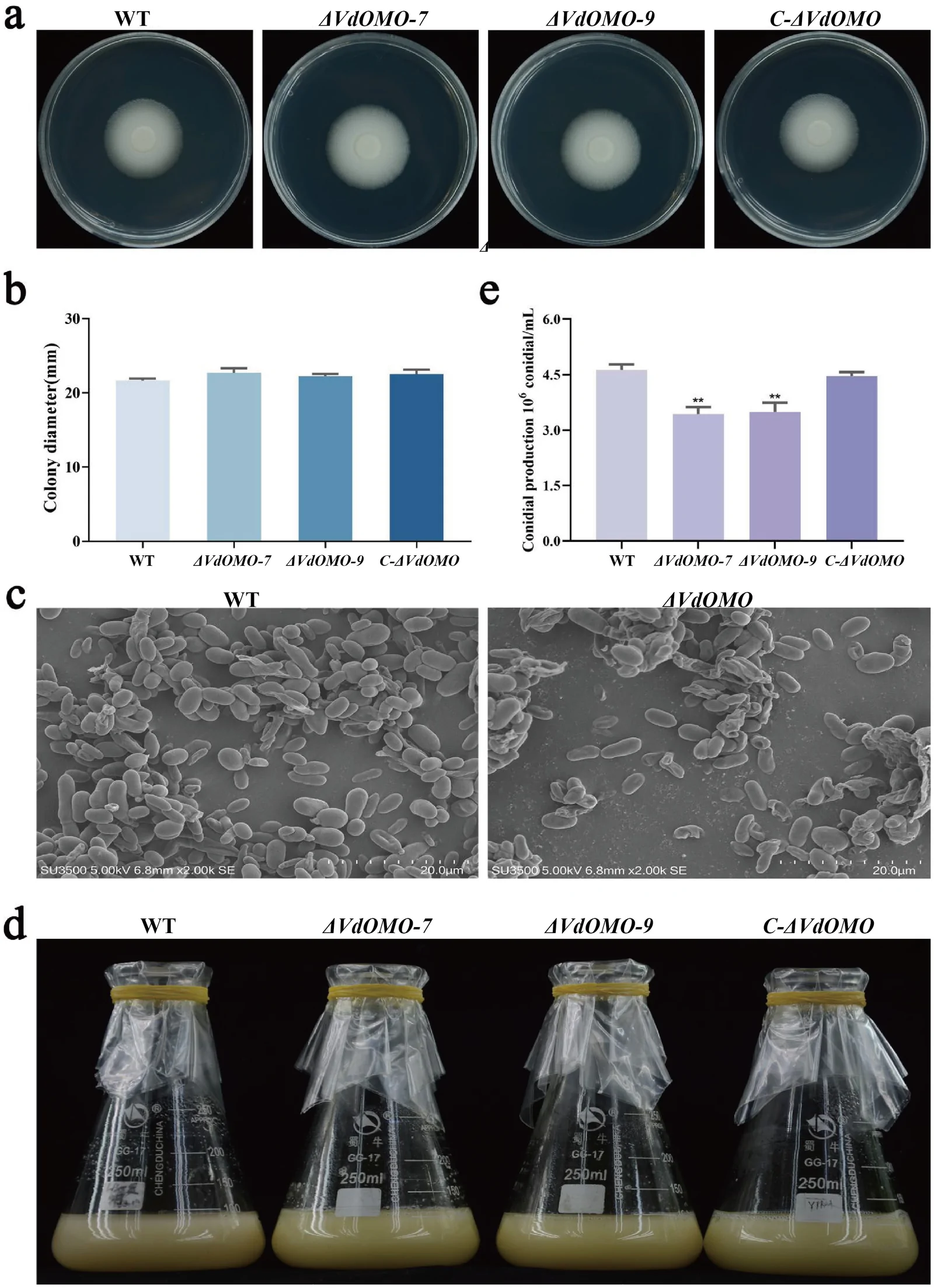
*VdOMO* contributes to conidial morphology and production in *V. dahliae*. **(a, b)** Representative colonies and colony diameters after growth on PDA at 26 °C for 7 ad. **(c)** Scanning electron micrographs of WT and *ΔVdOMO* conidia. Scale bar, 20 μm. **(d, e)** Representative appearance of the indicated strains after shaking incubation in PDB and the corresponding conidial yields. Data in **(b, e)** are mean ± SD (*n* = 3 independent biological replicates). Each dataset was analyzed separately by one-way ANOVA followed by Tukey’s multiple-comparison test. **P < 0.01.

Colony morphology and radial growth of WT, *ΔVdOMO-7*, *ΔVdOMO-9*, and *C-ΔVdOMO* strains were assessed on PDA after 7 ad of incubation. All strains exhibited comparable colony morphology and no significant differences in colony diameter (**Figures 3a, b**).

Scanning electron microscopy showed that WT conidia possessed smooth surfaces and uniform morphology. In contrast, some *ΔVdOMO* conidia displayed altered morphology, characterized by wrinkled or concave surfaces (**Figure 3c**). Furthermore, both *ΔVdOMO-7* and *ΔVdOMO-9* mutants produced significantly fewer conidia than the WT and complemented strains (**Figures 3d, e**). Consistently, the conidiation-related gene *VdNLP1* was significantly downregulated in the representative *ΔVdOMO* mutant, while *VdPLP* and *Vdpf* showed no significant expression differences (**Supplementary Figure 2**). These results collectively indicate that *VdOMO* plays a role in conidial morphology and conidial production in *V. dahliae*.

### 3.4 *VdOMO* influences microsclerotium formation and melanization

**Figure 4:**
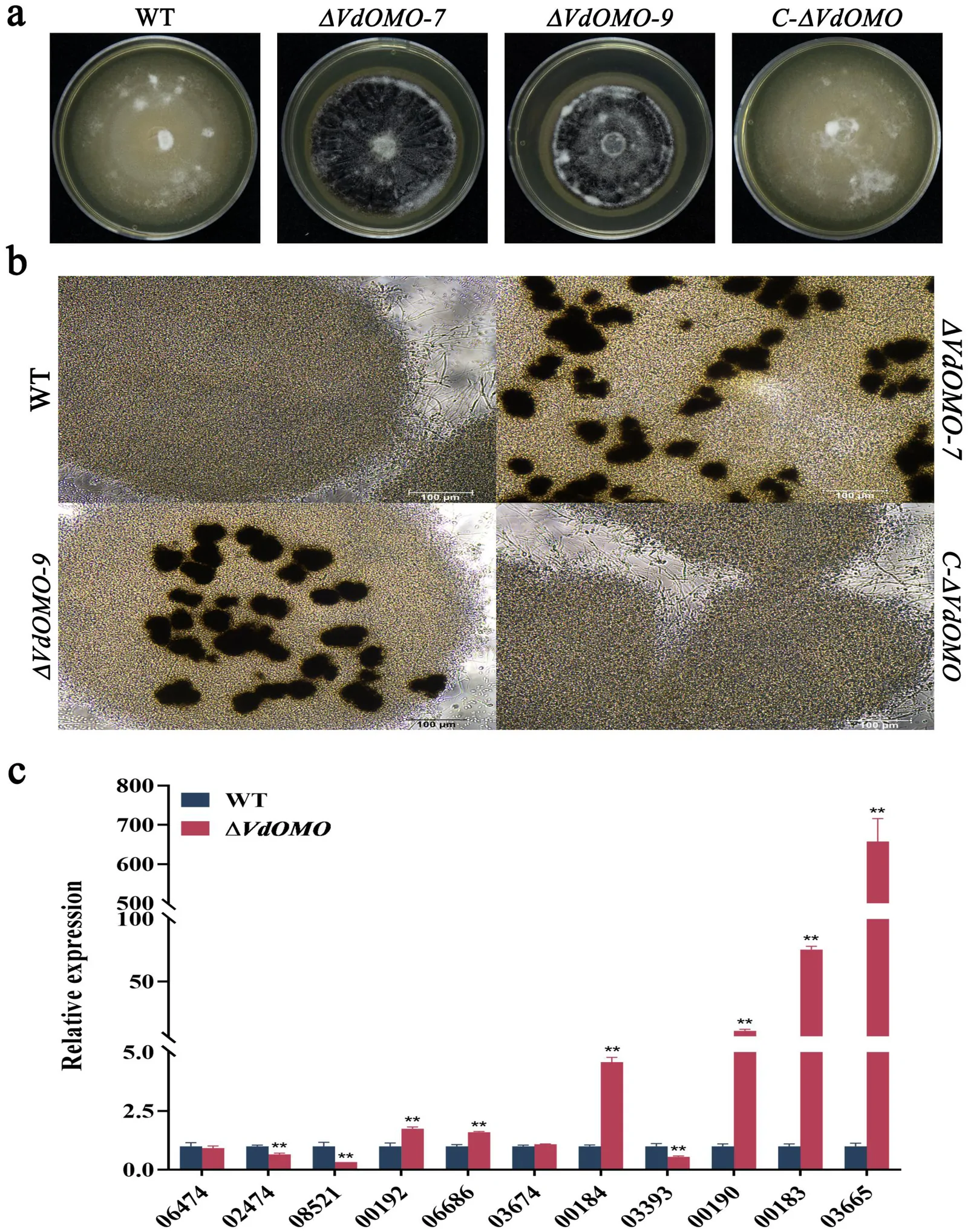
*VdOMO* influences microsclerotium formation and melanization. **(a)** Phenotypes of WT, *ΔVdOMO-7*, *ΔVdOMO-9*, and *C-ΔVdOMO* strains after one month of incubation on CM medium. **(b)** Microscopic observation of microsclerotium development in the indicated strains. Scale bar, 100 μm. **(c)** Relative transcript levels of microsclerotium- and melanization-associated genes. Data represent mean ± SD (*n* = 3 independent biological replicates). Each gene was analyzed separately using an unpaired two-tailed Student’s *t*-test based on ΔCt values. **P < 0.01.

After one month on complete medium (CM), the *ΔVdOMO-7* and *ΔVdOMO-9* mutants showed earlier and more extensive melanization compared to the WT and complemented strains (**Figure 4a**). Microscopic examination further revealed increased melanization of microsclerotia in the deletion mutants (**Figure 4b**). RT-qPCR analysis demonstrated significantly higher transcript levels of the melanin-associated genes *VDAG_00184*, *VDAG_00190*, *VDAG_00183*, and *VDAG_03665*, as well as the microsclerotium-associated gene *VDAG_00192*, in the representative *ΔVdOMO* mutant (**Figure 4c**). These findings collectively suggest that *VdOMO* plays a role in influencing both microsclerotium development and melanization in *V. dahliae*.

### 3.5 *VdOMO* contributes to growth on sucrose- and xylose-containing media

**Figure 5:**
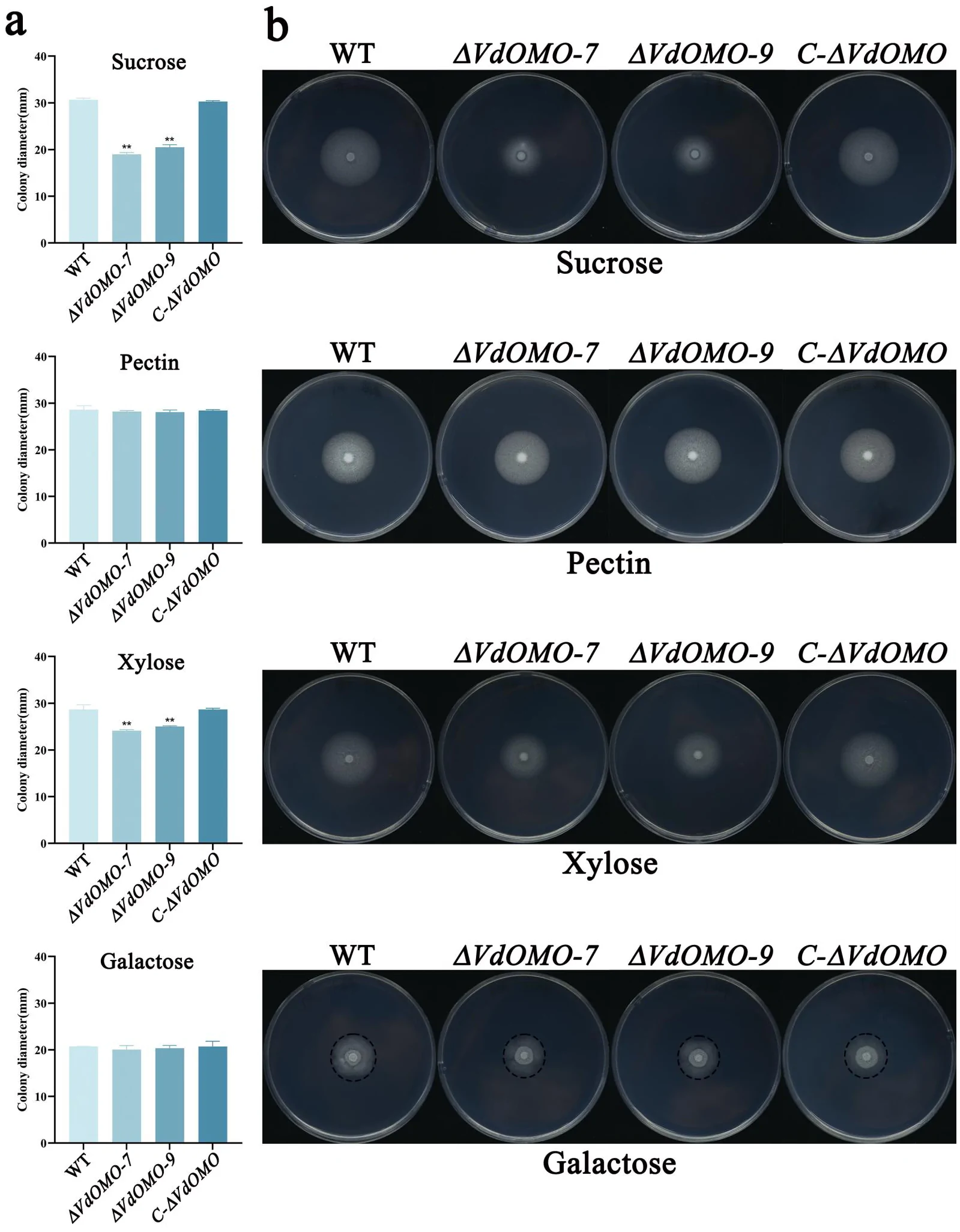
*VdOMO* affects growth on selected plant-related carbon sources. **(a)** Colony diameters of the WT, *ΔVdOMO-7*, *ΔVdOMO-9*, and *C-ΔVdOMO* strains. **(b)** Representative colonies grown on Czapek-Dox medium containing sucrose, pectin, xylose, or galactose as the sole carbon source at 26 °C for 7 ad. Data represent mean ± SD (*n* = 3 independent biological replicates) and were analyzed by two-way ANOVA followed by Tukey’s multiple-comparison test among strains within each carbon source. Only significant comparisons are shown. **P < 0.01.

To assess growth on plant-relevant carbon substrates, WT, *ΔVdOMO-7*, *ΔVdOMO-9*, and *C-ΔVdOMO* strains were cultured on Czapek-Dox media supplemented with sucrose, pectin, xylose, or galactose as the sole carbon source (**Figure 5**). The *ΔVdOMO* mutants showed significantly reduced colony diameters on sucrose- and xylose-containing media when compared to the WT and complemented strains. In contrast, no significant differences in growth were observed on pectin- or galactose-containing media. These results indicate that the deletion of *VdOMO* leads to carbon-source-dependent growth defects, rather than a general impairment across all tested carbon substrates.

### 3.6 *VdOMO* contributes to abiotic stress adaptation

**Figure 6:**
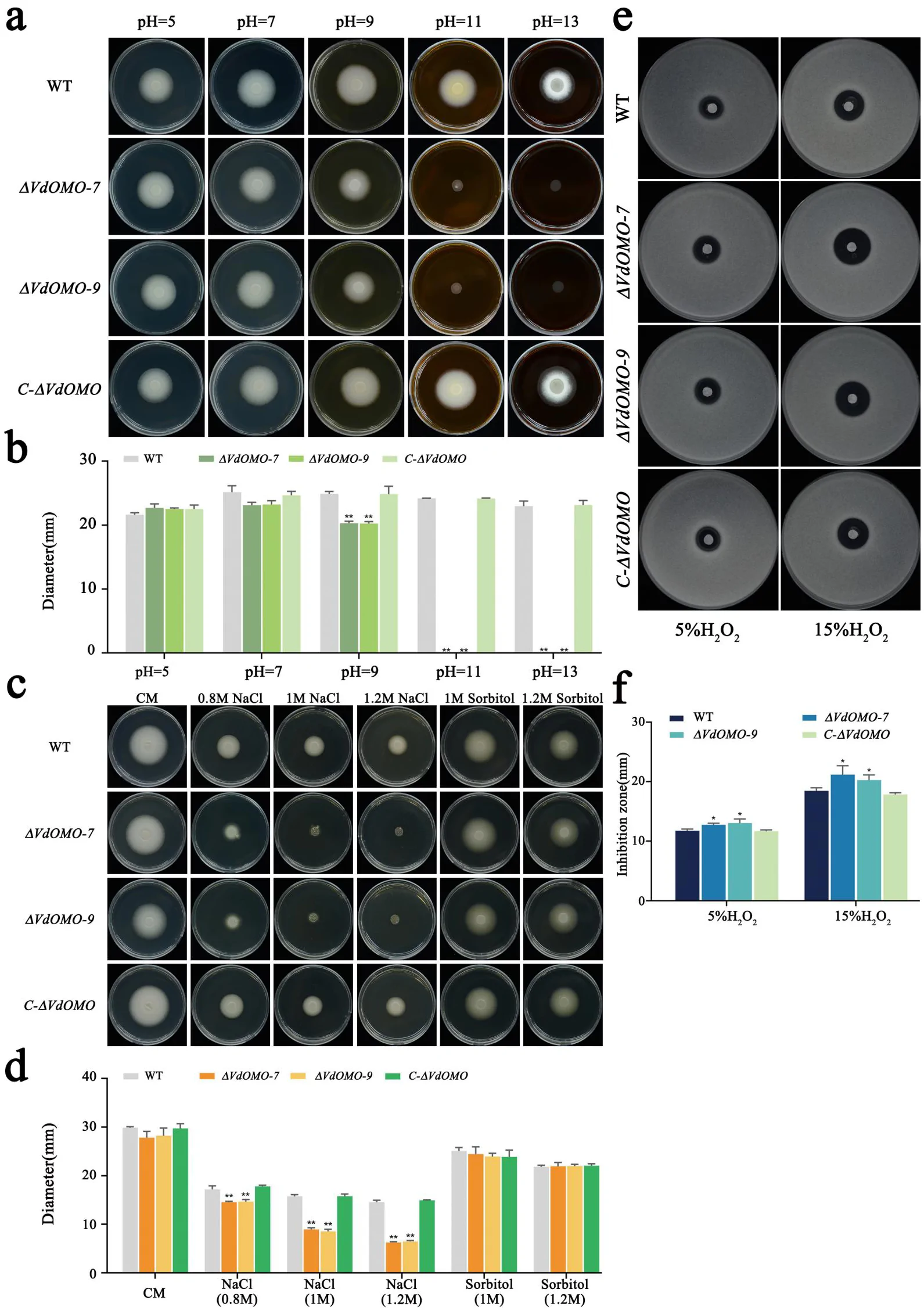
*VdOMO* contributes to abiotic stress adaptation. **(a, b)** Colony phenotypes and diameters under different pH conditions. (**c, d**) Colony phenotypes and diameters on CM supplemented with NaCl or sorbitol. **(e, f)** H_2_O_2_-induced inhibition zones and their diameters. Data in **(b, d, f)** represent mean ± SD (*n* = 3 independent biological replicates). Each dataset was analyzed separately by two-way ANOVA followed by Tukey’s multiple-comparison test among strains within each treatment. Only significant comparisons are shown. *P < 0.05; **P < 0.01.

The *ΔVdOMO* mutants displayed markedly reduced growth at pH 11 and 13 compared to the WT and complemented strains, indicating impaired adaptation to alkaline conditions (**Figures 6a, b**). Under osmotic stress, no significant differences were observed among strains on sorbitol-containing media. However, the *ΔVdOMO* mutants showed reduced growth under NaCl stress (**Figures 6c, d**). In addition, the *ΔVdOMO* mutants exhibited significantly larger inhibition-zone diameters under both 5% and 15% H_2_O_2_ treatments than the WT and complemented strains (**Figures 6e, f**). These results indicate that *VdOMO* plays a role in adaptation of *V. dahliae* to alkaline, salt, and oxidative stress conditions.

### 3.7 *VdOMO* contributes to cell wall integrity in *V. dahliae*

**Figure 7:**
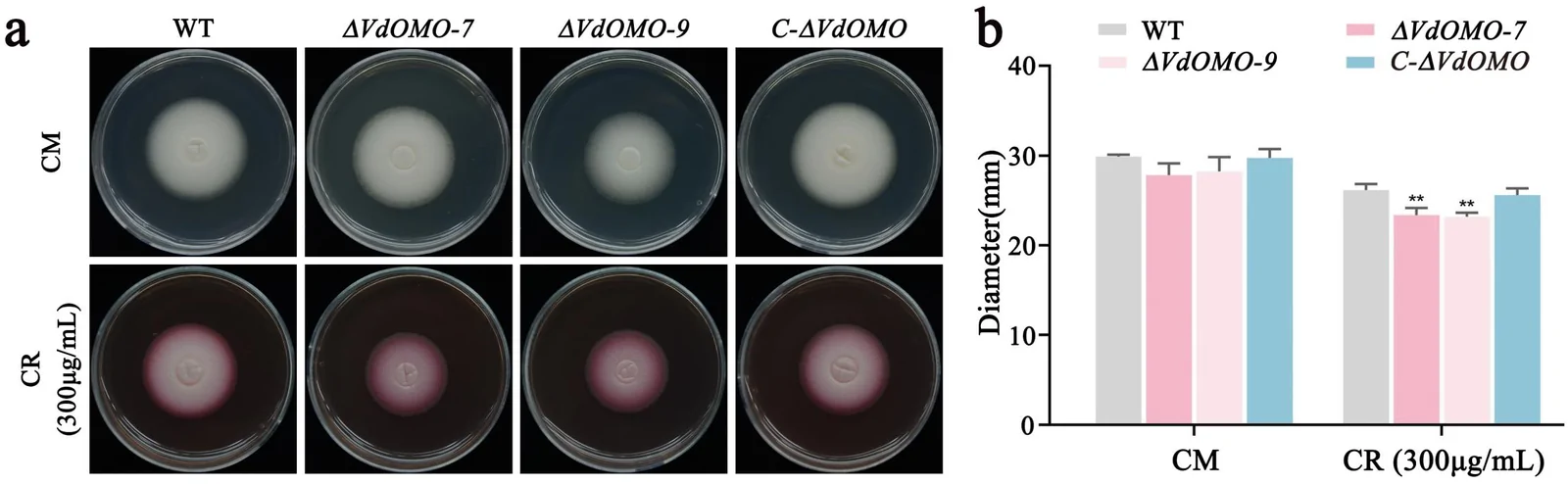
*VdOMO* contributes to cell wall integrity in *V. dahliae*. **(a)** Representative colonies of the WT, *ΔVdOMO-7*, *ΔVdOMO-9*, and *C-ΔVdOMO* strains grown on CM with or without 300 μg/mL Congo red at 26 °C for 7 ad. **(b)** Colony diameters under the indicated conditions. Data represent mean ± SD (*n* = 3 independent biological replicates) and were analyzed by two-way ANOVA followed by Tukey’s multiple-comparison test among strains within each treatment. Only significant comparisons are shown. **P < 0.01.

Colony growth was comparable across all strains on untreated CM. However, in the presence of 300 μg/mL Congo red, the *ΔVdOMO-7* and *ΔVdOMO-9* mutants exhibited significantly smaller colony diameters compared to the WT and complemented strains (**Figures 7a, b**). These results suggest that *VdOMO* contributes to cell wall integrity in *V. dahliae*.

### 3.8 *VdOMO* contributes to early colonization and mycelial penetration in *V. dahliae*

**Figure 8:**
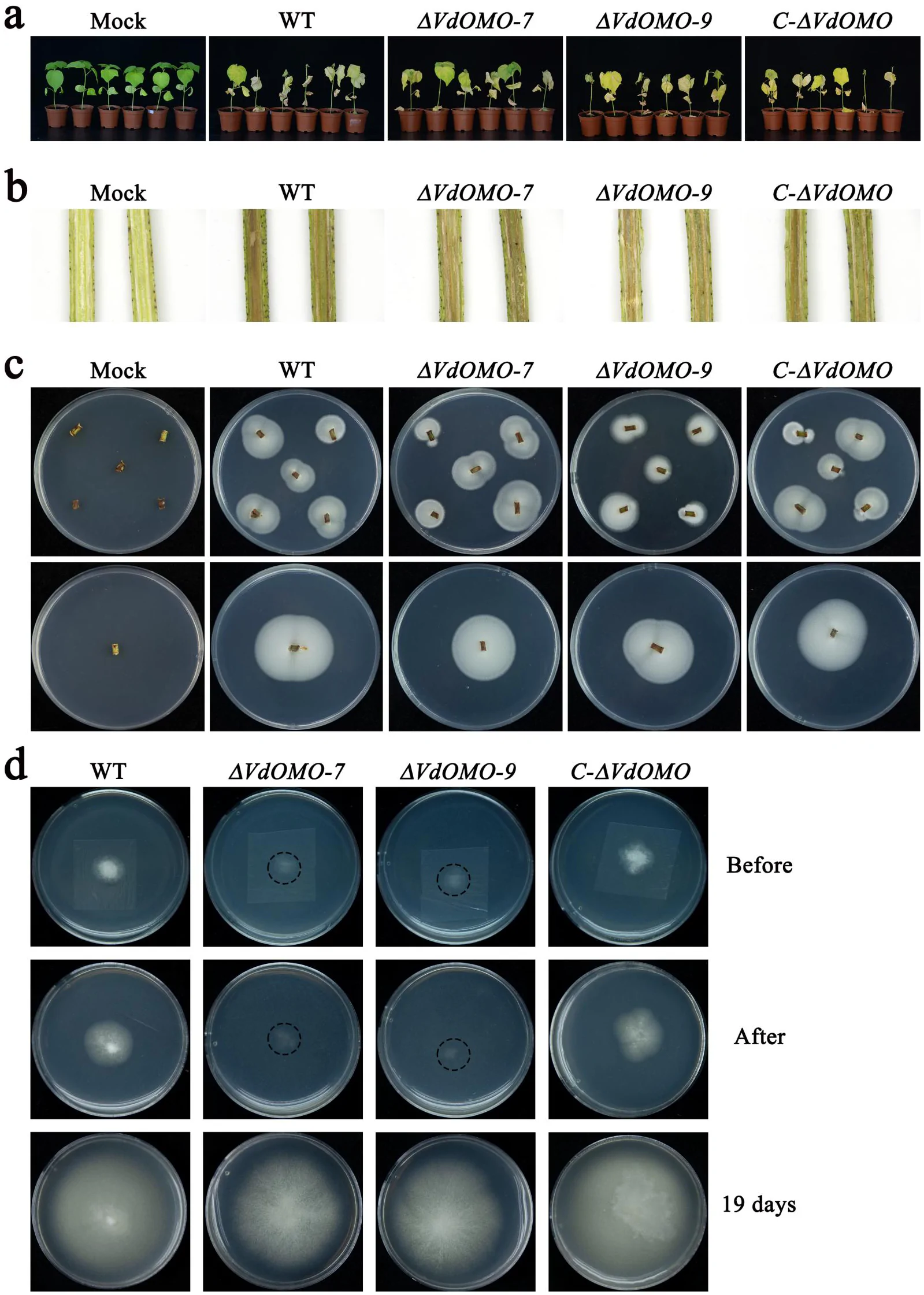
*VdOMO* contributes to early host colonization and mycelial penetration in *V. dahliae*. **(a)** Phenotypic observations of cotton plants inoculated with WT, *ΔVdOMO-7*, *ΔVdOMO-9*, and *C-ΔVdOMO* strains at 21 dpi. Mock, plants inoculated with sterile water. **(b)** Vascular browning in longitudinal sections of cotton seedling stems at 20 dpi. **(c)** Fungal recovery from cotton stem segments on PDA medium after inoculation with the indicated strains. **(d)** Cellophane penetration assay of WT, *ΔVdOMO-7*, *ΔVdOMO-9*, and *C-ΔVdOMO* strains. “Before” indicates growth on cellophane membranes for 3 ad; “After” indicates growth for 4 ad after removal of the cellophane membranes; “19 days” indicates colony growth after extended incubation for 19 days following removal of the cellophane membranes. Images are representative of three independent experiments. Quantitative fungal biomass data are shown in **Supplementary Figure 3a**.

Cotton plants were inoculated via wounded roots with the WT, *ΔVdOMO-7*, *ΔVdOMO-9*, and *C-ΔVdOMO* strains. At 21 dpi, all inoculated plants displayed leaf wilting and yellowing, with no significant differences in final visible disease symptoms among the inoculated groups (**Figure 8a**). Nevertheless, plants inoculated with the *ΔVdOMO* mutants displayed less severe vascular browning at 20 dpi compared to plants inoculated with the WT and complemented strains (**Figure 8b**). Fungal recovery from infected stem segments confirmed successful colonization by all strains (**Figure 8c**). Quantification of fungal biomass revealed that plants inoculated with the *ΔVdOMO* mutants accumulated significantly less fungal biomass than WT-inoculated plants at 9 and 15 dpi, although no significant difference was observed at 12 dpi (**Supplementary Figure 3a**). Furthermore, the deletion mutants demonstrated impaired cellophane penetration and exhibited time-dependent changes in the expression of penetration-associated genes (**Figure 8d**; **Supplementary Figures 3b, c**). Together, these results indicate that *VdOMO* contributes to early colonization- and penetration-related processes but is not a major determinant of final visible disease severity under the tested conditions.

### 3.9 Expression analysis of defense-related genes in cotton

**Figure 9:**
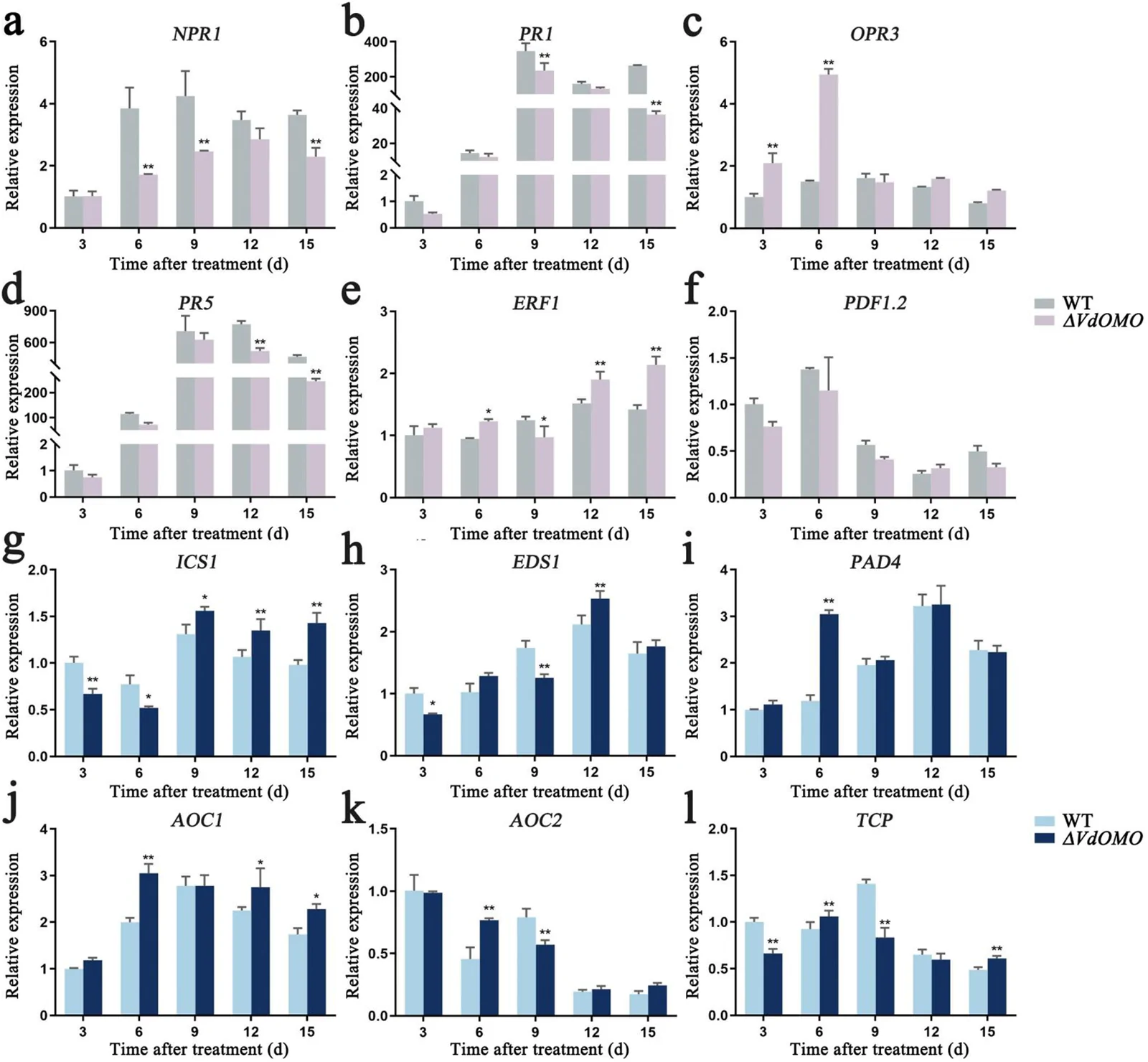
Expression profiles of defense-related genes in cotton. Transcript levels were measured at 3, 6, 9, 12, and 15 dpi: **(a)** *NPR1*. **(b)** *PR1*. **(c)** *OPR3*. **(d)** *PR5*. **(e)** *ERF1*. **(f)** *PDF1.2*. **(g)** *ICS1*. **(h)** *EDS1*. **(i)** *PAD4*. **(j)** *AOC1*. **(k)** *AOC2*. **(l)** *TCP*. Data are mean ± SD (*n* = 3 independent biological replicates). Each gene was analyzed separately by two-way ANOVA followed by Šídák’s multiple-comparison test between WT and *ΔVdOMO* at each time point. Statistical analyses were performed using ΔCt values. Only significant comparisons are shown. *P < 0.05; **P < 0.01.

At 15 dpi, cotton plants inoculated with the *ΔVdOMO* mutant exhibited significantly lower expression levels of *NPR1*, *PR1*, and *PR5* genes compared wth those inoculated with WT strain (**Figures 9a, b, d**). Conversely, at the same time point, the expression of *ICS1*, *AOC1*, and *TCP* genes was significantly higher in cotton plants inoculated with the *ΔVdOMO* strain than in those inoculated with WT (**Figures 9g, j, l**).

Several other genes showed transient or time-dependent differences at earlier infection stages, while *PDF1.2* showed no significant difference between treatments. These results indicate that cotton defense-related transcriptional responses dependent on both the fungal genotype and the infection stage.

### 3.10 *VdOMO* contributes to growth and conidial production under altered iron availability

**Figure 10:**
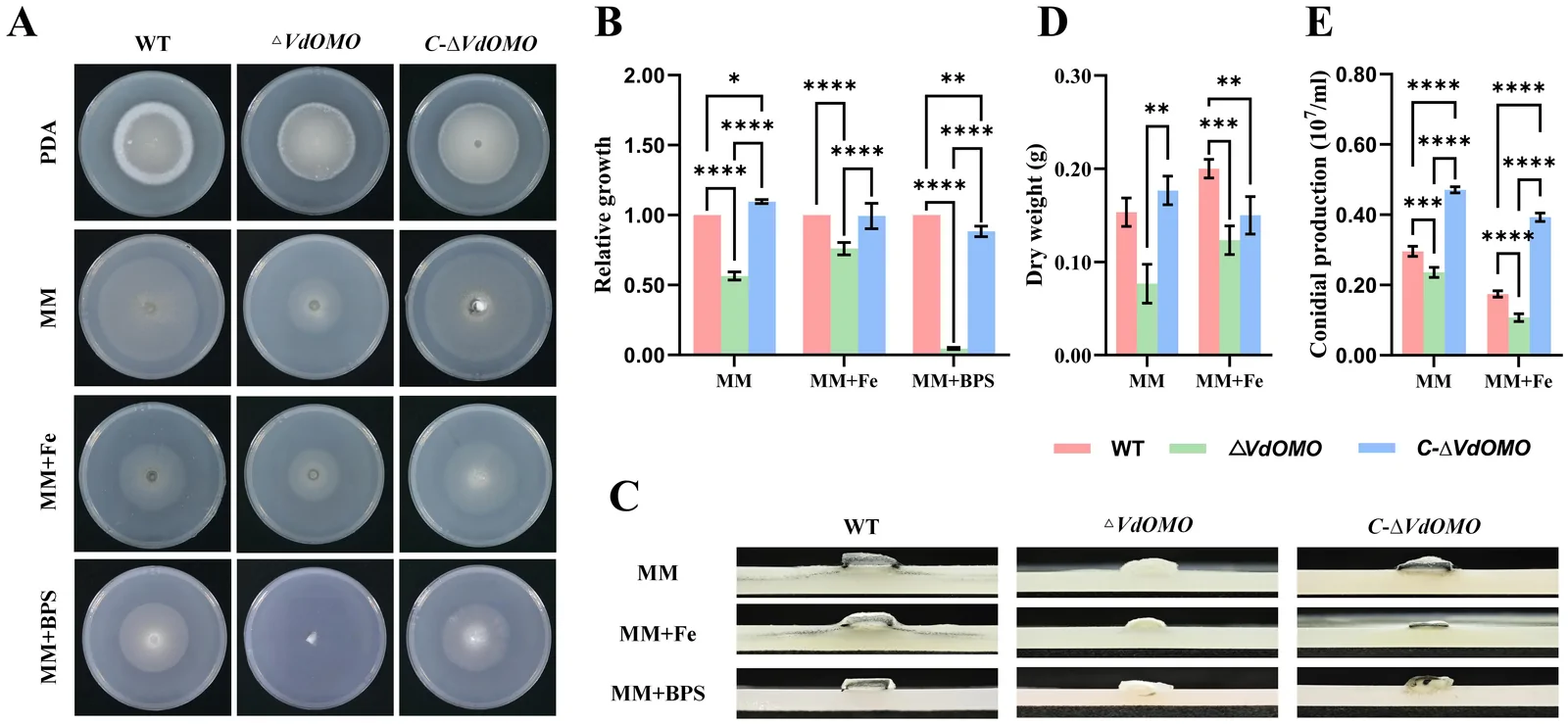
*VdOMO* supports fungal growth and conidial production under altered iron availability. **(A)** Colonies of the WT, *ΔVdOMO*, and *C-ΔVdOMO* strains grown on PDA, MM, MM+Fe (0.06 mM Fe), or MM+BPS (0.4 mM BPS) at 25 °C for 14 ad. **(B)** Relative colony growth, normalized to the WT within each MM-based condition. **(C)** Vertical colony sections under the indicated conditions. **(D)** Dry biomass after growth in liquid MM or MM+Fe for 14 ad. **(E)** Conidial yields after growth in liquid MM or MM+Fe for 7 ad. Data in **(B, D, E)** are mean ± SD (*n* = 3 independent biological replicates) and were analyzed separately by two-way ANOVA followed by Tukey’s multiple-comparison test among strains within each iron condition. Only significant comparisons are shown. *P < 0.05; **P < 0.01; ***P < 0.001; ****P < 0.0001.

The *ΔVdOMO* strain exhibited no significant radial-growth defect on PDA medium. However, its radial growth was significantly reduced on MM, MM+Fe, and MM+BPS media compared to the WT strain, with the most pronounced reduction observed under BPS-mediated iron limitation (**Figures 10A–C**). In liquid MM and MM+Fe, the dry biomass of the *ΔVdOMO* mutant was reduced to approximately 50% and 62% of the WT levels, respectively (**Figure 10D**). Conidial production was also significantly reduced under both conditions. In MM, the mutant produced approximately 79% of the WT conidial yield, whereas a more substantial reduction was observed in MM+Fe (**Figure 10E**). The complemented strain largely restored radial growth, biomass, and conidial production, although the extent of recovery varied depending on the culture conditions and measured traits. These results indicate that *VdOMO* contributes to fungal growth and conidial production under conditions of altered iron availability.

### 3.11 Transcriptional association among *VdOMO*, *VDAG_05314*, and *VdASP*

**Figure 11:**
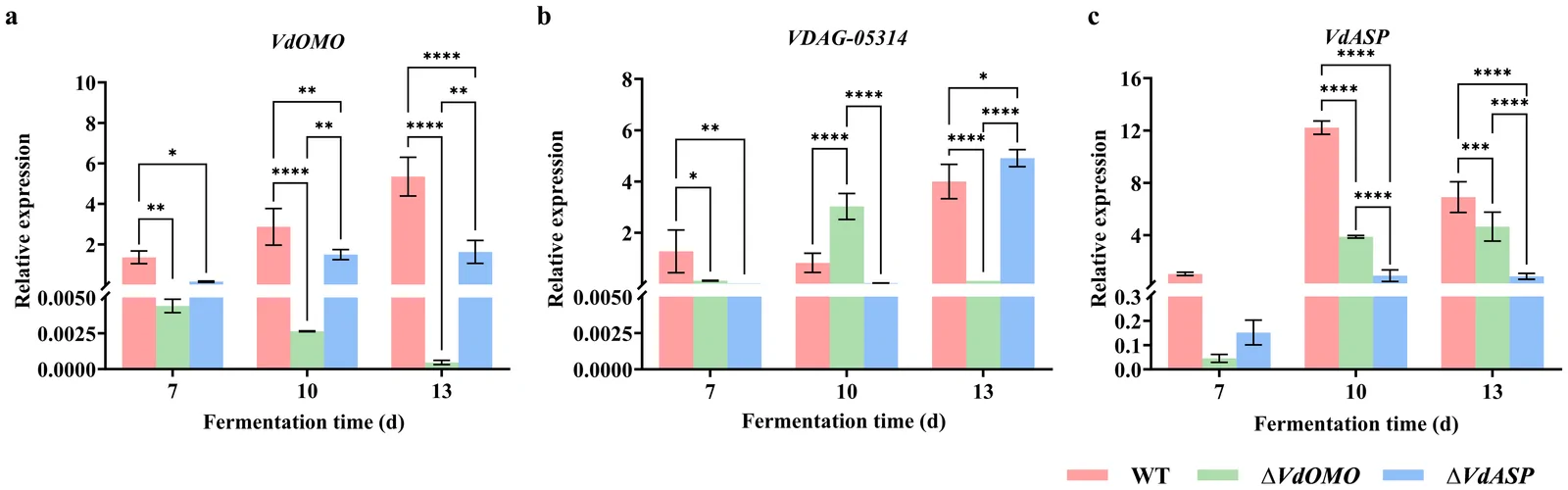
Time-course expression of *VdOMO*, *VDAG_05314*, and *VdASP*. Relative transcript levels of **(a)** *VdOMO*, **(b)** *VDAG_05314*, and **(c)** *VdASP* in the WT, *ΔVdOMO*, and *ΔVdASP* strains after 7, 10, and 13 ad of cultivation. Data are mean ± SD (*n* = 3 independent biological replicates). Each gene was analyzed separately by two-way ANOVA followed by Tukey’s multiple-comparison test among strains within each time point. Statistical analyses were performed using ΔCt values. Only significant comparisons are shown. *P < 0.05; **P < 0.01; ***P < 0.001; ****P < 0.0001.

*VdOMO* transcript levels gradually increased in the WT strain over time but remained extremely low in the *ΔVdOMO* strain, confirming the loss of *VdOMO* expression in the deletion mutant. In the *ΔVdASP* strain, *VdOMO* transcript levels were lower than those in WT at all tested time points (**Figure 11a**). The expression pattern of *VDAG_05314* varied among strains and growth stages. *VDAG_05314* expression was elevated in the *ΔVdOMO* strain at day 10, whereas at day 13, higher transcript levels were observed in the WT and *ΔVdASP* strains than in the *ΔVdOMO* strain (**Figure 11b**). *VdASP* transcript levels were markedly increased in the WT strain at days 10 and 13, whereas *VdASP* expression was lower in the *ΔVdOMO* and *ΔVdASP* strains (**Figure 11c**). Collectively, these results reveal genotype- and growth-stage-dependent expression patterns among *VdOMO*, *VDAG_05314*, and *VdASP*. However, these results do not establish direct co-regulation, promoter-level regulation, or physical interaction among these genes.

### 3.12 *VdOMO* contributes to extracellular CAS-reactive iron-chelating activity, intracellular iron accumulation, and condition-dependent iron-responsive gene expression

**Figure 12:**
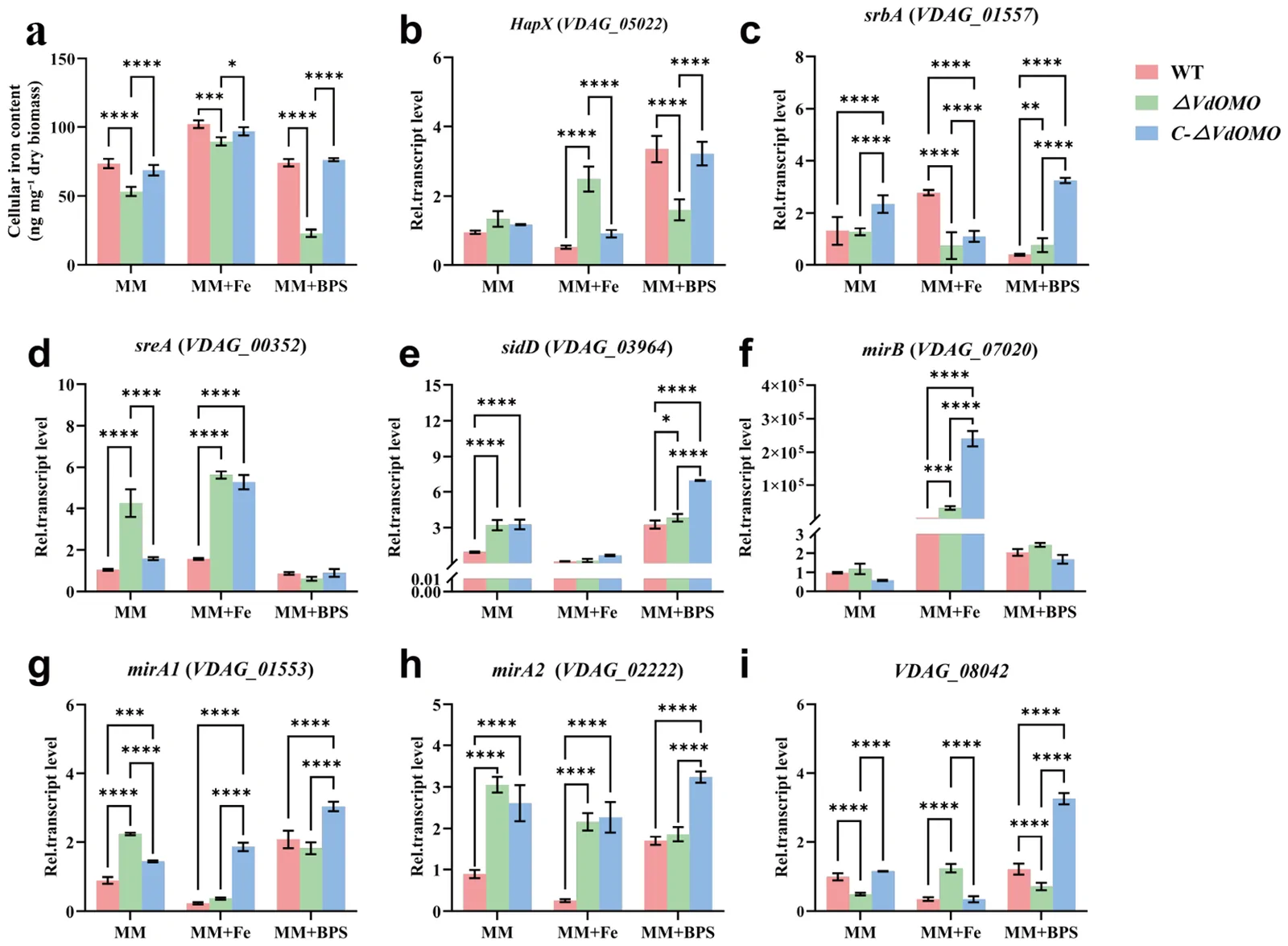
Intracellular iron accumulation and expression of iron-related genes under different iron-availability conditions. **(a)** Cellular iron content of the WT, *ΔVdOMO*, and *C-ΔVdOMO* strains cultured in MM, MM+Fe, or MM+BPS. Values were normalized to mycelial dry biomass. **(b–i)** Relative transcript levels of **(b)** *HapX*; **(c)** *srbA*; **(d)** *sreA*; **(e)** *sidD*; **(f)** *mirB*; **(g)** *mirA1*; **(h)** *mirA2*; and **(i)** *VDAG_08042*. Data are mean ± SD (*n* = 3 independent biological replicates). Each panel was analyzed separately by two-way ANOVA followed by Tukey’s multiple-comparison test among strains within each iron condition. Transcript data were analyzed using ΔCt values. Only significant comparisons are shown. A discontinuous y-axis is used in **(f)**. *P < 0.05; **P < 0.01; ***P < 0.001; ****P < 0.0001.

To quantify extracellular CAS-reactive iron-chelating activity, the WT, *ΔVdOMO*, and *C-ΔVdOMO* strains were cultured in low-iron liquid MM and analyzed using a liquid CAS shuttle assay. Mycelial dry biomass did not differ significantly among strains (**Supplementary Figure 4A**). After normalization to dry biomass, the *ΔVdOMO* mutant exhibited significantly lower extracellular CAS-reactive iron-chelating activity than WT, whereas the complemented strain showed a level comparable to WT (**Supplementary Figure 4B**). Cellular iron content was subsequently quantified under MM, MM+Fe, and MM+BPS conditions. Two-way ANOVA revealed significant effects of genotype, iron condition, and their interaction. The *ΔVdOMO* mutant accumulated significantly less intracellular iron than WT under all three conditions, with the greatest reduction observed under BPS-mediated iron limitation. In each condition, the complemented strain recovered to a level not significantly different from WT (**Figure 12a**).

Expression profiling further revealed condition-dependent transcriptional responses to *VdOMO* deletion (**Figures 12b–i**). Under MM conditions, *sreA* expression was approximately 4.0-fold higher in the *ΔVdOMO* mutant than in WT, while *sidD* and *mirA2* increased by approximately 3.4-fold and *mirA1* by 2.5-fold. In contrast, *VDAG_08042* was significantly downregulated, whereas *HapX*, *srbA*, and *mirB* showed no significant changes. Under Fe-supplemented conditions, *HapX*, *sreA*, *mirB*, *mirA2*, and *VDAG_08042* were upregulated by approximately 4.8-, 3.6-, 11.3-, 8.9-, and 3.5-fold, respectively, whereas *srbA* expression decreased to 26.4% of the WT level; *sidD* and *mirA1* remained unchanged. Under BPS-mediated iron limitation, *srbA* expression increased by approximately 2.0-fold and *sidD* showed a modest but significant increase to approximately 1.1 times the WT level. Conversely, *HapX* and *VDAG_08042* expression decreased to approximately 50% and 60% of the WT levels, respectively, while *sreA*, *mirB*, *mirA1*, and *mirA2* showed no significant differences. Several of these transcriptional changes were partially or fully restored in the complemented strain. Collectively, *VdOMO* deletion reduced extracellular CAS-reactive iron-chelating activity and intracellular iron accumulation and was associated with condition-dependent transcriptional changes in iron-related genes.

## 4 Discussion

In this study, *VdOMO* was found to influence several key processes in *V. dahliae*, including conidial development, microsclerotium formation and melanization, stress adaptation, carbon-source-dependent growth, and cell wall integrity. The observation of earlier melanization and microsclerotia in the *ΔVdOMO* mutants, coupled with altered transcription of associated genes, suggests that *VdOMO* plays a role in regulating the timing of resting-structure development. Given that microsclerotia and DHN melanin are crucial for the long-term survival of *V. dahliae* in soil, this accelerated phenotype may reflect a shift in developmental or metabolic balance rather than a simple activation of the melanin pathway (Xiong et al., 2019; Harting et al., 2020). However, direct regulation of DHN-melanin biosynthetic genes by *VdOMO* was not investigated. Beyond these developmental effects, deletion of *VdOMO* led to increased sensitivity to alkaline pH, NaCl, and H_2_O_2_, whereas growth under sorbitol stress remained unaffected. Furthermore, selective growth defects were observed on sucrose- and xylose-containing media, but not on pectin- or galactose-containing media. This suggests that *VdOMO* contributes to specific environmental and nutrient responses rather than causing a general reduction in growth capacity. As sucrose is a major soluble carbohydrate in plant tissues and xylose is a common component of hemicellulosic cell-wall polysaccharides, the reduced growth of the mutants on these substrates may indicate selective limitations in utilizing plant-associated nutrients (Koch, 2004; Cosgrove, 2005; Voragen et al., 2009).

While final visible disease symptoms did not significantly differ between plants inoculated with the WT and *ΔVdOMO* strains under the tested conditions, the mutants exhibited weaker vascular browning and accumulated less fungal biomass at specific infection stages. These findings, along with impaired cellophane penetration and altered expression of penetration-associated genes, suggest that *VdOMO* contributes to early colonization and penetration processes. However, it does not appear to be a major determinant of final visible disease severity in this assay (Zhao et al., 2016; Bui et al., 2019). Additionally, cotton plants inoculated with the *ΔVdOMO* mutants displayed altered expression of several defense-related genes. Nevertheless, without measurements of phytohormone contents and signaling activities, these transcriptional differences cannot definitively establish changes in SA-, JA-, or ET-mediated signaling or mechanistically explain the observed colonization phenotype.

Transcriptional profiling of *VdOMO*, *VDAG_05314*, and *VdASP* revealed growth-stage-dependent expression changes among the three genes. *VdOMO* and *VdASP* transcript levels changed concomitantly in the corresponding deletion strains at specific time points, whereas *VDAG_05314* displayed a strain- and growth-stage-dependent pattern. These observations indicate an expression-level association among the three genes located in the same genomic region, but they do not establish direct transcriptional regulation, physical interaction, or a specific regulatory hierarchy.

Phylogenetic and pairwise sequence analyses classified VdOMO as a putative SidA-family L-ornithine N5-monooxygenase. It is important to note that proteins designated SidA, Sid1, Sib2, or OMO1 in various fungal species are homologous members of this enzyme family, not amino acid-identical proteins. This classification aligns with the established function of fungal L-ornithine N5-monooxygenases in hydroxamate-type siderophore biosynthesis (Schrettl et al., 2004; Hissen et al., 2005; Pecoraro et al., 2021). Functionally, *VdOMO* deletion reduced biomass-normalized extracellular CAS-reactive iron-chelating activity under low-iron conditions and decreased intracellular iron accumulation, with the strongest effect under BPS-mediated iron limitation. Iron-related genes also showed distinct, condition-dependent transcriptional responses, indicating that the consequences of *VdOMO* deletion varied with iron availability rather than adhering to a uniform regulatory pattern. Together, these findings support the contribution of *VdOMO* to siderophore-associated iron acquisition and iron-responsive processes. However, it is crucial to acknowledge certain limitations. The CAS assay measures total extracellular iron-chelating capacity and does not identify individual siderophore molecules. Moreover, ferric reductase activity, direct siderophore profiling, and promoter- or DNA-binding activity were not examined. Consequently, the present study does not establish direct regulation of iron-related genes or define the precise mechanism linking *VdOMO* to intracellular iron status.

## 5 Conclusion

In summary, the VdOMO protein is a putative SidA-family L-ornithine N5-monooxygenase that contributes to fungal development, stress adaptation, siderophore-associated iron acquisition, and early host colonization in *V. dahliae*. While *VdOMO* deletion impaired fungal biomass accumulation at selected infection stages and cellophane penetration, it did not appreciably alter final visible disease severity under the tested conditions. These findings provide a foundational basis for further elucidating the precise mechanisms by which *VdOMO* links iron acquisition with fungal development and host colonization.

## Data Availability

The original contributions presented in the study are included in the article/**Supplementary Material.** Further inquiries can be directed to the corresponding authors.
