# Supplementary material for: VdOMO contributes to development, stress adaptation, siderophore-associated iron acquisition, and early host colonization in Verticillium dahliae
Source: Front Plant Sci. 2026 Aug 19;17:1905175. doi: 10.3389/fpls.2026.1905175 (PMC13533957; doi:10.3389/fpls.2026.1905175)
Supplement: Supplementary file 1 [file DataSheet1.pdf]

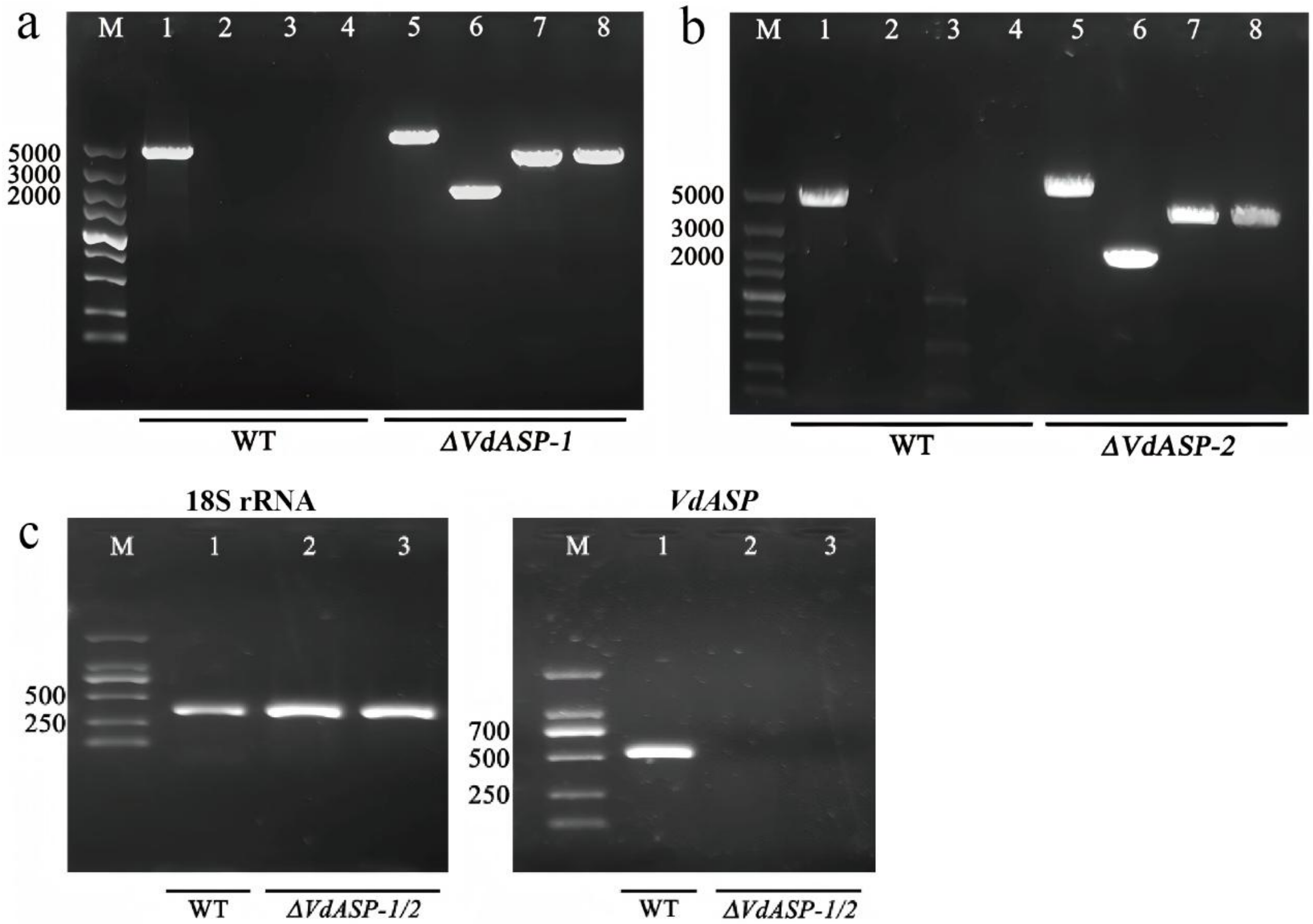

Fig. S1. Molecular validation of the *VdASP* deletion mutants. (a, b) Diagnostic PCR analyses of WT and *ΔVdASP-1* or *ΔVdASP-2*, respectively. M, DNA marker; lanes 1-4, WT; lanes 5-8, the corresponding *ΔVdASP* mutant. Lanes 1 and 5, locus-spanning PCR; lanes 2 and 6, *hph*-specific PCR; lanes 3 and 7, 5′ recombination-junction PCR; lanes 4 and 8, 3′ recombination-junction PCR. (c) RT-PCR detection of *VdASP* transcripts. Lanes 1–3 represent WT, *ΔVdASP-1*, and *ΔVdASP-2*, respectively. The 18S rRNA amplicon served as the cDNA-quality control. *VdASP* transcripts were detected in WT but not in either deletion mutant.

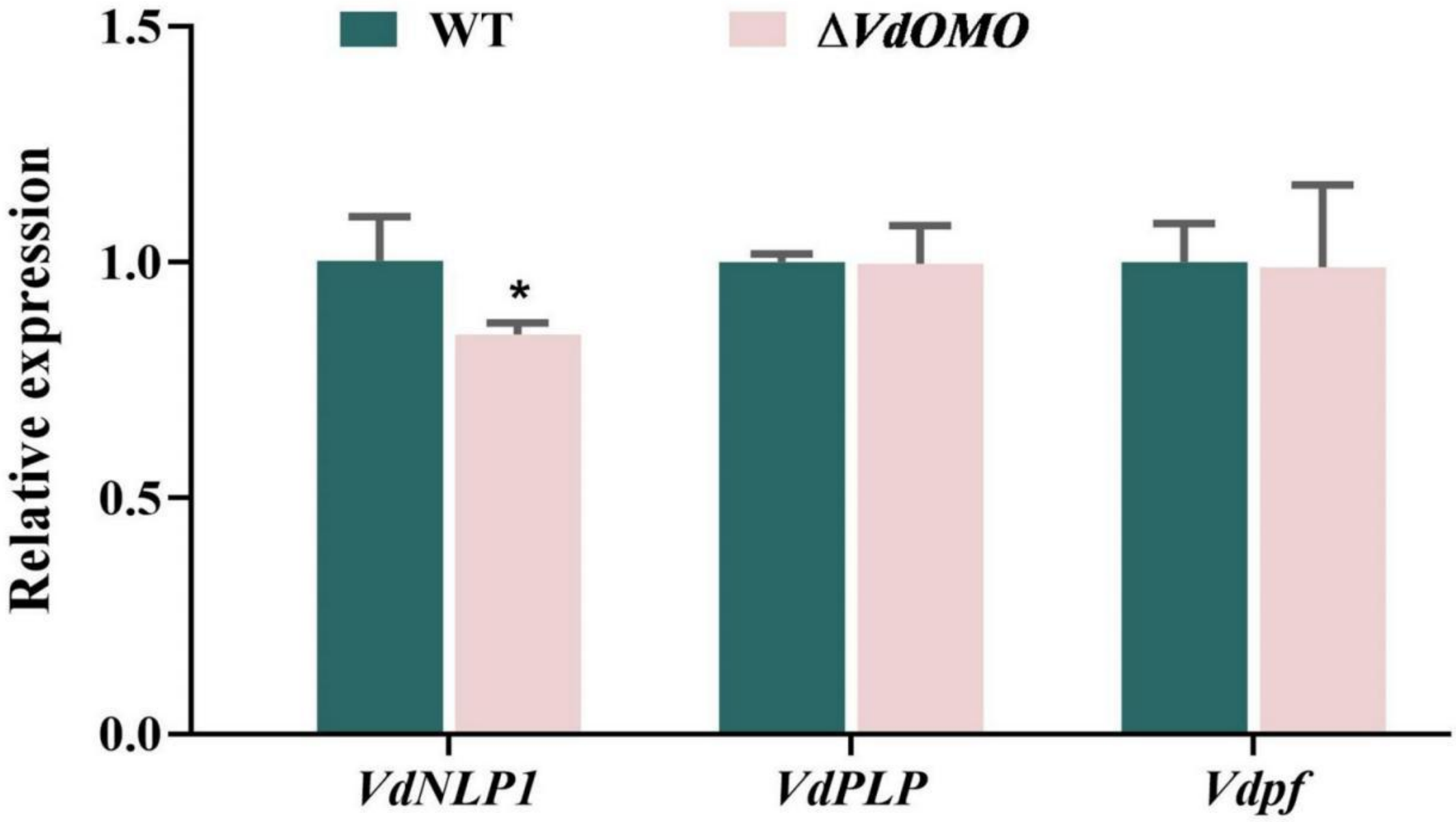

Fig. S2. Expression of conidiation-related genes in the WT and *ΔVdOMO* strains. The relative expression levels of *VdNLP1*, *VdPLP*, and *Vdpf* were determined by RT-qPCR. Data are presented as mean ± SD from three independent biological replicates. Each gene was analyzed separately using an unpaired two-tailed Student's *t*-test comparing the WT and *ΔVdOMO* strains. Statistical analyses were performed using ΔCt values. *, $P < 0.05$.

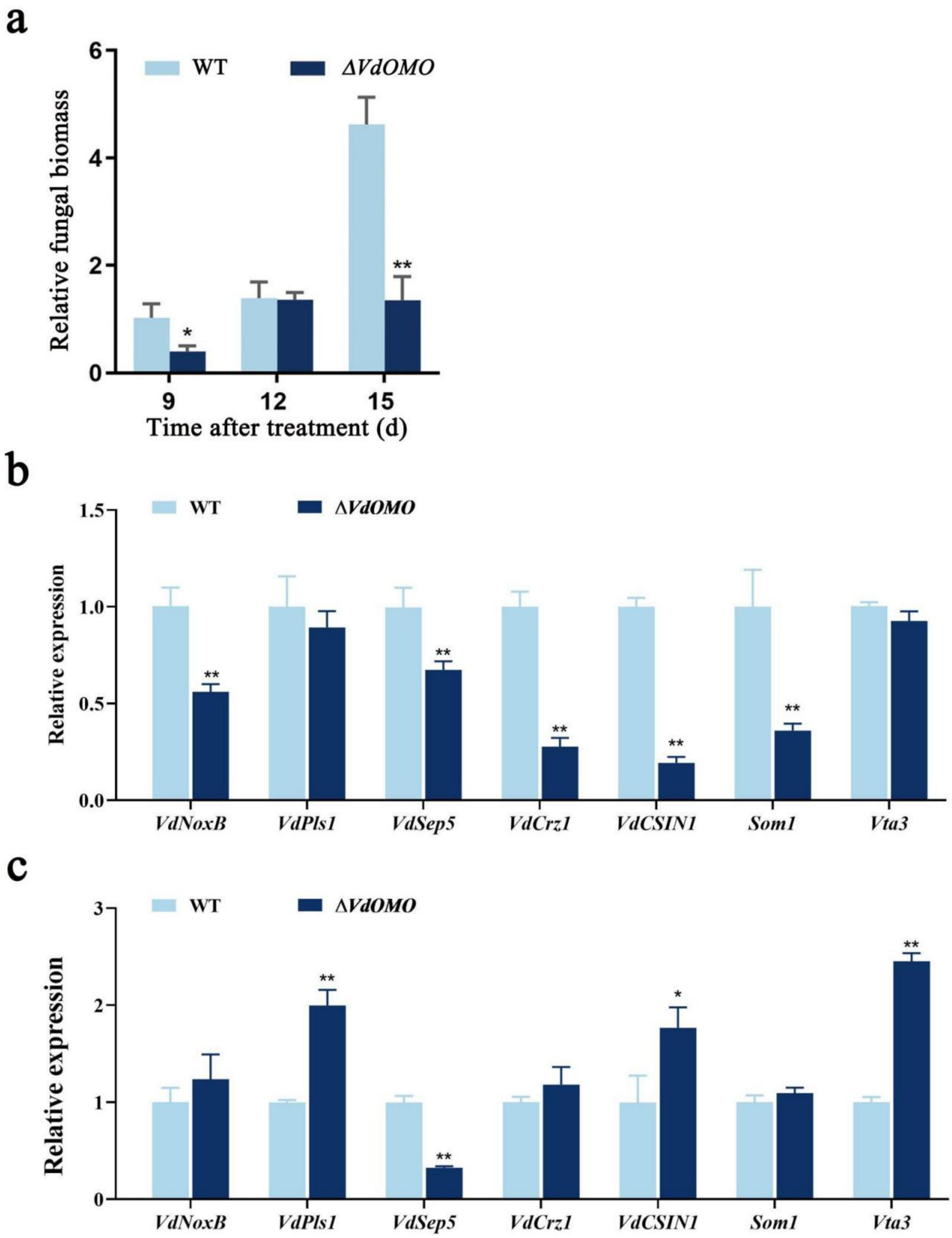

Fig. S3. Fungal biomass quantification and expression analysis of genes associated with mycelial penetration. (a) Quantification of *V. dahliae* biomass in cotton roots. (b) Relative expression levels of infection-related genes in the WT and *ΔVdOMO* strains after 5 d of growth. (c) Relative expression levels of infection-related genes in the WT and *ΔVdOMO* strains after 10 d of growth. Data are presented as mean ± SD from three independent biological replicates. Fungal biomass in panel (a) was analyzed using two-way ANOVA, with genotype and time as factors, followed by Šídák's multiple-comparison test comparing the WT and *ΔVdOMO* strains at each time point. In panels (b) and (c), each gene was analyzed separately using an unpaired two-tailed Student's *t*-test comparing the WT and *ΔVdOMO* strains. Statistical analyses of RT-qPCR data were performed using ΔCt values. Only significant comparisons are indicated. *, $P < 0.05$; **, $P < 0.01$.

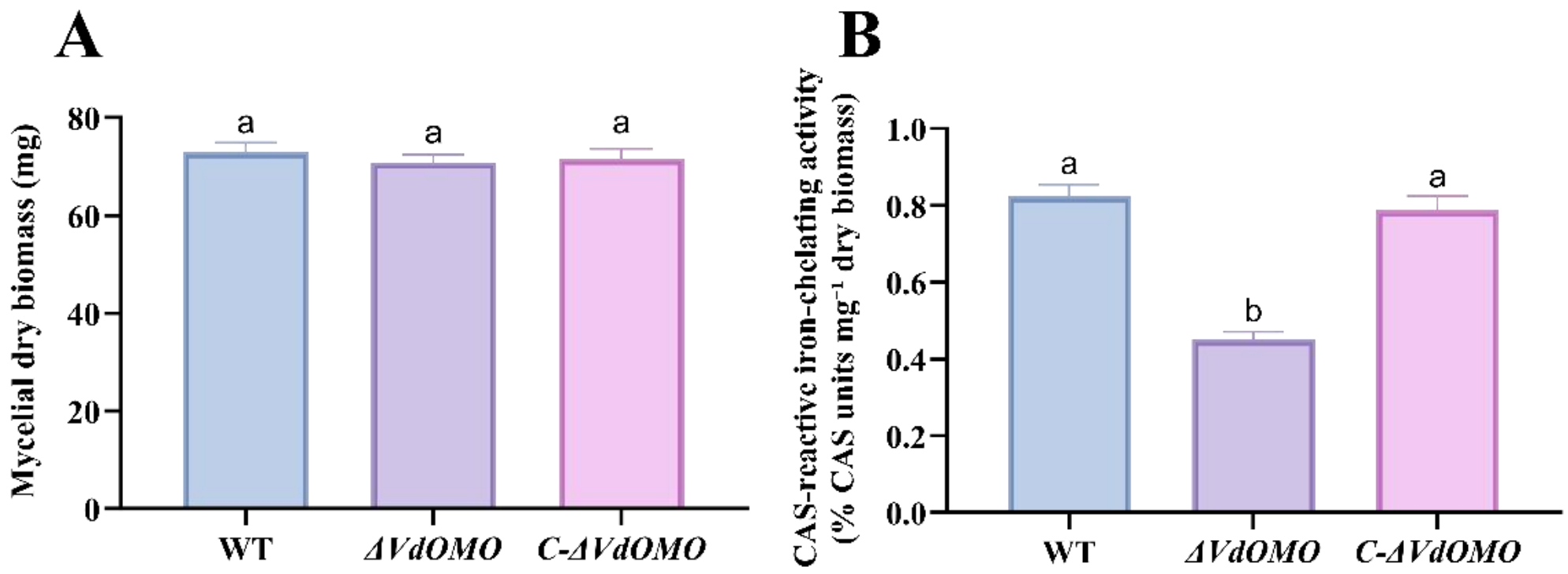

Fig. S4. Quantitative assessment of extracellular CAS-reactive iron-chelating activity. (A) Mycelial dry biomass of Vd991 (WT), *ΔVdOMO*, and *C-ΔVdOMO* after cultivation in low-iron liquid MM at 25°C and 200 rpm for 72 h. (B) Biomass-normalized extracellular CAS-reactive iron-chelating activity determined using a liquid CAS shuttle assay. CAS-reactive activity was calculated as percent CAS units and normalized to mycelial dry biomass. Data are presented as mean ± SD from three independent biological replicates. Three technical measurements were averaged to obtain one value for each biological replicate. Panels (A) and (B) were analyzed separately using one-way ANOVA followed by Tukey's multiple-comparison test. Different lowercase letters indicate significant differences among strains at $P < 0.05$.

Table S1. Pairwise amino acid sequence conservation between VdOMO and representative fungal SidA/Sid1/Sib2/OMO1 homologs.

Species	Protein	Accession	Length (aa)	Aligned residue pairs	VdOMO coverage (%)	Homolog coverage (%)	Identity (%)	Similarity (%)
Verticillium alfalfae	SidA-like	XP_002999718.1	515	492	98.50	100.00	98.58	99.19
Sodiomyces alkalinus	SidA-like	XP_028471626.1	582	523	99.25	99.31	69.79	82.60
Fusarium lateritium	SidA-like	KAM0343510.1	543	504	97.94	99.63	63.49	77.38
Fusarium oxysporum	SidA-like	KAJ9423647.1	521	476	97.94	99.62	64.08	76.89
Fusarium verticillioides	SidA-like	XP_018748703.1	547	499	97.94	99.63	64.93	78.96
Fusarium pseudograminearum	SidA	EKJ70529.1	536	492	97.94	99.63	65.65	79.47
Trichoderma asperelloides	SidA-like	KAH8126866.1	546	510	98.32	99.63	59.41	74.90
Simplicillium lamellicola	SidA-like	KAO0488644.1	520	492	97.94	99.42	61.99	75.81
Hypoxylon trugodes	SidA-like	XP_051312882.1	518	473	92.34	96.91	63.00	77.17
Colletotrichum chlorophyti	SidA-like	OLN95217.1	553	517	99.81	99.82	68.86	82.98
Colletotrichum karsti	SidA-like	XP_038750010.1	546	512	99.81	99.82	69.14	83.40
Pyricularia oryzae	OMO1	EHA47238.1	563	512	98.32	99.82	62.70	75.98
Aspergillus fumigatus	SidA	E9QYP0	501	429	88.41	95.01	56.18	70.86
Aspergillus nidulans	SidA	XP_663427.1	498	427	86.54	90.96	55.27	70.26
Aspergillus clavatus	SidA	XP_001267710.1	501	437	88.60	95.01	53.09	68.19
Aspergillus steynii	SidA	PLB47033.1	502	425	86.92	90.44	54.12	70.35
Beauveria bassiana	SidA	J4W6X9	536	499	97.94	99.25	58.72	73.95
Beauveria brongniartii	SidA	OAA38195.1	536	499	97.94	99.25	59.12	74.15
Schizosaccharomyces pombe	Sib2	Q9P7T0	431	387	86.92	97.22	42.64	62.53
Ustilago maydis	Sid1	P56584	649	452	90.84	90.45	38.72	60.84

Table S2. Primer sequences used for PCR and RT-PCR validation.

Primer name	Primer sequence (5'-3')
VdOMO-5OUT	CCGAAGATAACCCCGAATTC
VdOMO-5F	AGAAGAGTAATCTAGAAGGGGCCCCTGCAATCCAG
VdOMO-5R	TTAGTGAGGGTTAATTGCGCGATGAGCGGGAATTAATGAC
VdOMO-3F	AGTTGTTCCCAGTGATCTTCGAACTCGATCTTCAACCCCTC
VdOMO-3R	ATTACGAATTGGATCCGACGGGCGATGCATCAGTC
VdOMO-3OUT	GGCGTCAATGTTGTCTCTTC
hyg-F	GCGCAATTAACCCTCACTAAA
hyg-R	CGAAGATCACTGGGAACAACT
VdOMOCom-F	GCCCCTGGGTTGAATTCTGCAGATTCTTCAAGCCCTT
VdOMOCom-R	TCGACTCTAGAGGATCCTCTTTCGAGCACCGACTGA
18S rRNA-F	CCGCCGGTCCATCAGTCTCTCTGTTTATA
18S rRNA-R	CGCCTGCGGGACTCCGATGCGAGCTGTAAC
VdOMOYZ-F	TCGTTCATCAAGGACATGGC
VdOMOYZ-R	CTCTTCGGAATCAGAGGCCGTC
VdASP-5OUT	AGTGGAACACCGATCCCTTC
VdASP-5F	AGAAGAGTAATCTAGGTACCTCTACGGCCGAGGTG
VdASP-5R	TTAGTGAGGGTTAATTGCGCGACGGCAGGTCTTAATCTAT
VdASP-3F	AGTTGTTCCCAGTGATCTTCGCTGGAACCGGCAATGGGGAC
VdASP-3R	ATTACGAATTGGATCCGTTGCTCGACCCATCTCTC
VdASP-3OUT	ATCACCCTCCTGACGGCTTC

Table S3. Primer sequences used for RT-qPCR analysis.

Gene Name	Primer sequence (5'-3')
VdNLP1	F: TCGGTCTTTGCCCTCGTC; R: GCCTGGTTTGCGTTGTTC
VdPLP	F: GCTGACCAGTATCTGTCGGAGG; R: ATGACGACTGGCTTCTCGGCCT
Vdpf	F: ACCATTTTCAACAGTCGGGTACGCG;<br>R: GTGTGACGTACCAGCAACCGCTT
VDAG_06474	F: AGAAGCGGACACGAGACA; R: GGGTGAACAGATACGGGATA
VDAG_02474	F: GGCTGCGACCTCTCTCTTAC; R: TACTTGTGCGCCCTGATCTC
VDAG_08521	F: AGAAGATGACAAGGCGTGGG; R: GCTACGAGGATGAAGGTCGG
VDAG_00192	F: GCCGGACAAGGCAATATGC; R: CCAATGTATAGGCACCAAGACTGAT
VDAG_06686	F: ACAAGACTCTCCGCCTTTGG; R: GAGCTCCCAAACCTTGACGA
VDAG_03674	F: ACCGTCATGGAGATGTACGC; R: TGCGAGTACACAGGCAGAAG
VDAG_00184	F: ACAAGCCGTTGTTCTATCACATCA;<br>R: GCTGTCACCACGTCTGATAGATC
VDAG_03393	F: AAAGGTGTTTGAGAGCGGAC; R: ATCTCCCTCTCCACAACAGC
VDAG_00190	F: ACCGCTCCTACATCCG; R: CACGCCGACTTCCTTA
VDAG_00183	F: TATGTCCCTGGCGGCTTTAA; R: TGATCCACTCGCAGTCTTCA
VDAG_03665	F: TCGTGAACTACGCCAACT; R: TGACGTGCCCGAAAGA
GhUBQ7	F: GAAGGCATTCCACCTGACCAAC;<br>R: CTTGACCTTCTTCTTCTTGTTGCTTG
ITS	F: TCCGTAGGTGAACCTGGGG; R: TCCTCCGCTTATTGATATGC
VdNoxB	F: TGCGTGGCAAGCATAAGACATAC;<br>R: GACAGCACGAGTGAAATCACCAAC
VdPls1	F: ATGGTCAACAAGATCCTCGCGA; R: TCCGGCTGCTCAAACATGTTGT
VdCrz1	F: ATGGATCAGCAAGCTCAACATCG; R: GATCCAGACCGAGACCGAGAC
VdSep5	F: AGCTCGACCTGGACGAGGA; R: GAGGCTTCGTTATCAATCTCGTCTC
VdCSIN1	F: CTTTGATTGTGGTATGGGTTCT; R: GTGGTGGGTTTGCCTTGT
Vta3	F: GATGTCTGCCCTGCGTAA; R: GATCTGAGCCTGGTCAAAGT
Som1	F: GTCGTGACAACCGAAAGCAG; R: TCCCTCGTGGAGCGCAAA
Histone3	F: GCCAAGCGTGTCACAATTATGC;<br>R: ACATCACATTGAACCTACCACTACC
NPR1	F: CTAGCTTGCGGAGGGATTGATACC;<br>R: GAGATGGCTGACCTGTCAAACTGC
PR1	F: AAGAATGTGGGTTAGTGAGAGGGT;<br>R: ACCACTTGAGTATAATGCCCGC
OPR3	F: TGGAAGATGATTGTGGATGCTG; R: GAGATGGGTTTGTTTGTTGAGGA
PR5	F: AAGGAGTCCACCACAATCACCG; R: CTGGCACTGCTATGGCTCGTAT
ERF1	F: CCAAGTTCCGACAATGTTATGC; R: CCAACAGCTCGTCCGACAA

PDF1.2	F: CTGTGGTAGCGGATGGTGATAAG;<br>R: GTGCAGACGCATTTGCGAAGGAA
ICS1	F: ATGGATGAATGGGTGCGAAGG;<br>R: AAGAATGCCAGAGGTAAGAGGAGGA
EDS1	F: GCAGCAACAGCTCCTCTACCTCAA;<br>R: GGCAGACCAAGACGCTACAGATACA
PAD4	F: GGATGGAAGAATGGAAAGAAATGAA;<br>R: GAACTAGGAAAGCAGACTAAGGAACCA
AOC1	F: CTCCCAAGCCCACAAAAGTT; R: ACAGAGTCCAGCCGTGATGC
AOC2	F: ATCTACTTCCACTCTTCCT; R: GCCATTCCTTCTTCTTCTA
TCP	F: CATCCGAACCCCAGATATGG; R: TGTAAGGGTGCTTGCAATGTG
VdOMO	F: TCGTTCATCAAGGACATGGC; R: CTCTTCGGAATCAGAGGCCGTC
VdASP	F: TCAACGAGCTAGAGGCCTCAGT; R: ATCCCCGTCATCTTCCCACTCG
VDAG_05314	F: TGACCACCCCACCCTCACTCTTTTCT;<br>R: AGGGCGAAGCCAGATACTCTCATAGT
VDAG_05022	F: CTTCTCATCATCCCGACCTCA; R: TCTTGGGCAACTCCGACTG
VDAG_01557	F: AGAGCAAGCAAACGACGATG; R: GGATGATGTGGCAGTTCAAGAC
VDAG_00352	F: GCCGCCGATAACGATAGTCT; R: CCTCTTCGCTCTGTGATTGC
VDAG_03964	F: TACCGCGAGAATGACGATGG; R: GCTGATGTCAAGGAGCAGGT
VDAG_07020	F: TGGTGCCCAGCAAGAATG; R: CCCGTTGGTTAGGAAGAGG
VDAG_01553	F: GGGCGATATGCGAAGAATG; R: CGATCAAGCAGACCGACA
VDAG_02222	F: AGTGTCTACCGTCCGTTCCT; R: GATGAATGGCTTTGTCTGCTTGAA
VDAG_08042	F: ATTGCTCGCTGTTTGCTGTG; R: TGGATGTTGCCCTTCTCGTC
β-tubulin	F: TTTCCAGATCACCCACTCC; R: ACGACCGAGAAGGTAGCC
